# Dbo/Henji Modulates Synaptic dPAK to Gate Glutamate Receptor Abundance and Postsynaptic Response

**DOI:** 10.1371/journal.pgen.1006362

**Published:** 2016-10-13

**Authors:** Manyu Wang, Pei-Yi Chen, Chien-Hsiang Wang, Tzu-Ting Lai, Pei-I Tsai, Ying-Ju Cheng, Hsiu-Hua Kao, Cheng-Ting Chien

**Affiliations:** 1 Institute of Molecular Medicine, National Taiwan University, Taipei, Taiwan; 2 Institute of Molecular Biology, Academia Sinica, Taipei, Taiwan; 3 Institute of Neuroscience, National Yang-Ming University, Taipei, Taiwan; New York University, UNITED STATES

## Abstract

In response to environmental and physiological changes, the synapse manifests plasticity while simultaneously maintains homeostasis. Here, we analyzed mutant synapses of *henji*, also known as *dbo*, at the *Drosophila* neuromuscular junction (NMJ). In *henji* mutants, NMJ growth is defective with appearance of satellite boutons. Transmission electron microscopy analysis indicates that the synaptic membrane region is expanded. The postsynaptic density (PSD) houses glutamate receptors GluRIIA and GluRIIB, which have distinct transmission properties. In *henji* mutants, GluRIIA abundance is upregulated but that of GluRIIB is not. Electrophysiological results also support a GluR compositional shift towards a higher IIA/IIB ratio at *henji* NMJs. Strikingly, dPAK, a positive regulator for GluRIIA synaptic localization, accumulates at the *henji* PSD. Reducing the *dpak* gene dosage suppresses satellite boutons and GluRIIA accumulation at *henji* NMJs. In addition, dPAK associated with Henji through the Kelch repeats which is the domain essential for Henji localization and function at postsynapses. We propose that Henji acts at postsynapses to restrict both presynaptic bouton growth and postsynaptic GluRIIA abundance by modulating dPAK.

## Author Summary

To meet various developmental or environmental needs, the communication between pre- and postsynapse can be modulated in different aspects. The release of presynaptic vesicles can be regulated at the steps of docking, membrane fusion and endocytosis. Upon receiving neurotransmitter stimuli from presynaptic terminals, postsynaptic cells tune their responses by controlling the abundance of different neurotransmitter receptors at the synaptic membrane. The *Drosophila* NMJ is a well-defined genetic system to study the function and physiology of synapses. Two types of glutamate receptors (GluRs), IIA and IIB, present at the NMJ, exhibit distinct desensitization kinetics: GluRIIA desensitizes much slower than GluRIIB does, resulting in more ionic influx and larger postsynaptic responses. By altering the ratio of GluRIIA to GluRIIB, muscle cells modulate their responses to presynaptic release efficiently. However, how to regulate this intricate GluRIIA/GluRIIB ratio requires further study. Here, we describe a negative regulation for dPAK, a crucial regulator of GluRIIA localization at the PSD. Henji specifically binds to dPAK near the postsynaptic region and hinders dPAK localization from the PSD. By negatively controlling dPAK levels, synaptic GluRIIA abundance can be restrained within an appropriate range, protecting the synapse from unwanted fluctuations in synaptic strengths or the detriment of excitotoxicity.

## Introduction

Coordinated action and communication between pre- and postsynapses are essential in maintaining synaptic strength and plasticity. Presynaptic strength or release probability of synaptic vesicles involves layers of regulation including vesicle docking, fusion, and recycling, as well as endocytosis and exocytosis. Also, how postsynapses interpret the signal strength from presynapses depends largely on the abundance of neurotransmitter receptors at the synaptic membrane [1, 2]. During long-term potentiation, lateral diffusion of extrasynaptic α-amino-3-hydroxy-5-methyl-4-isoxazolepropionic acid receptor (AMPAR) to synaptic sites is accelerated [3, 4] and the exocytosis of AMPAR is enhanced near the postsynaptic density (PSD), causing an accumulation of synaptic receptors [5, 6]. In contrast, under the long-term depression condition, synaptic AMPAR is reduced by hastened endocytosis [7, 8]. While molecular mechanisms are proposed to play roles in regulating and fine-tuning postsynaptic glutamate receptor (GluR) abundance in plasticity models, the developmental regulation of GluR abundance at the synaptic surface still needs to be elucidated.

Synapses at the *Drosophila* neuromuscular junction (NMJ) use glutamate as the neurotransmitter, and have properties reminiscent of mammalian central excitatory synapses [9, 10]. Homologous to vertebrate AMPAR and kainate receptors, *Drosophila* GluR subunits assemble as tetramers to gate ion influx [11]. Each functional receptor contains essential subunits (GluRIIC, GluRIID and GluRIIE) and either GluRIIA or GluRIIB; therefore, synaptic GluRs can be classified according to their subunit compositions as either A- or B-type receptors [12–17]. These two types of receptors exhibit distinct developmental and functional properties. Newly-formed PSDs tend to accumulate more GluRIIA channels, while the IIA/IIB ratio becomes more balanced when PSDs mature [18]. In addition, GluRIIB channels have much faster desensitization kinetics, which results in smaller quantal size than GluRIIA channels [12]. Therefore, the synaptic composition of these two types of GluRs greatly influences the postsynaptic interpretation of neuronal activities. The *Drosophila* homolog of p21-activated kinase (dPAK) regulates GluRIIA abundance at the PSD; GluRIIA receptor clusters at the postsynaptic membrane are strongly reduced in *dpak* mutants [19]. However, overexpression of dPAK in postsynapses is not sufficient to increase GluRIIA cluster size, suggesting that dPAK activity in regulating GluRIIA abundance is tightly controlled.

Ubiquitination and deubiquitination play critical roles in regulating synaptic functions [20–24]. In loss-of-function mutants for *highwire*, a gene encoding a conserved E3 ubiquitin ligase, NMJs overgrow, producing supernumerary synaptic boutons [25, 26]. This phenotype is duplicated by overexpression of the deubiquitinating enzyme Fat facets (Faf) in presynapses [27]. These studies underline the importance of balanced ubiquitination in synapse formation and function. Cullin-RING ubiquitin ligases (CRLs) are large protein complexes that confer substrate ubiquitination [28, 29]. Importantly, CRLs promote ubiquitination through substrate receptors that provide specific recognition of substrates for ubiquitination. The BTB-Kelch proteins are suggested to be the substrate receptors for Cul3-scaffolded CRLs [30–32].

In this study, we identified a BTB-Kelch-containing protein, Henji, also known as Dbo [33], which regulates NMJ growth and synaptic activity by restricting the clustering of GluRIIA. Synaptic size of *henji* mutants was significantly expanded, as viewed under transmission electron microscopy (TEM). Immunostaining for dPAK and GluRIIA also suggests larger areas of PSDs in the absence of Henji, and the intensity of each fluorescent punctum becomes stronger, indicating abnormal accumulation of these PSD proteins. By genetically reducing one gene dosage of *dpak* in *henji* mutants, GluRIIA accumulation and abnormal bouton morphology was suppressed. In contrast, reducing the *gluriia* gene dosage in *henji* mutants restored bouton morphology but failed to suppress dPAK accumulation. Thus, Henji regulates bouton morphology and GluRIIA clustering levels likely through a control of dPAK. Interestingly, while overexpression of dPAK, either constitutively active or dominantly negative, had no effects on GluRIIA clustering, overexpression of these dPAK forms in *henji* mutants modulated GluRIIA levels, indicating that Henji limits the action of dPAK to regulate GluRIIA synaptic abundance. Henji localized to the subsynaptic reticulum (SSR) surrounding synaptic sites, consistent with the idea that Henji functions as a gatekeeper for synaptic GluRIIA abundance.

## Results

### Altered bouton morphology and GluR composition in *henji* mutants

We are interested in how ubiquitination might regulate synaptic function through controlling specific synaptic proteins. As putative substrate receptors of Cul3-based E3 ubiquitin ligases, each BTB-Kelch protein could recognize one or multiple synaptic proteins to regulate their abundance and thus their synaptic functions [31]. The *Drosophila* genome encodes 16 BTB-Kelch proteins (S1 Fig) and those with available RNAi or P-element insertion lines were examined for NMJ morphological abnormality. By immunostaining for horseradish peroxidase (HRP) and Synapsin to reveal NMJ morphology, we found the P-element insertion (*PBac{PB}dbo*^*c04604*^) in the *CG6224* locus induced satellite boutons (see below). Whereas *CG6224* is known to encode Dbo [34], given the supernumerary bouton morphology, we named this mutant “*henji*”, meaning “very crowded” in Mandarin, and this name is used in this study.

To study Henji function *in vivo*, we generated mutants by P-element excision in the annotated *CG6224/dbo* locus (S2A Fig). Two excision mutants with truncation of the shared promoter region between *CG6224* and *CG6169* were lethal. Addition of the *CG6169* genomic transgene rescued the lethality of these deletions, which are named *henji*^*1*^ and *henji*^*8*^ with disruption specifically in *henji* expression (S2A Fig). Indeed, the *henji* mRNA expressions analyzed by reverse transcription PCR (RT-PCR) showed lower levels in all three *henji* mutants used in this study, with a medium level in the *PBac{PB}dbo*^*c04604*^ insertion line (named as *henji*^*P*^ in this study), a low level in *henji*^*1*^, and an almost undetectable level in *henji*^*8*^ (S2B Fig). As the protein translation start site was deleted in *henji*^*8*^, we conclude that *henji*^*8*^ is a null allele, *henji*^*1*^ a strong loss-of-function allele, and *henji*^*P*^ a hypomorphic allele.

**Fig 1:**
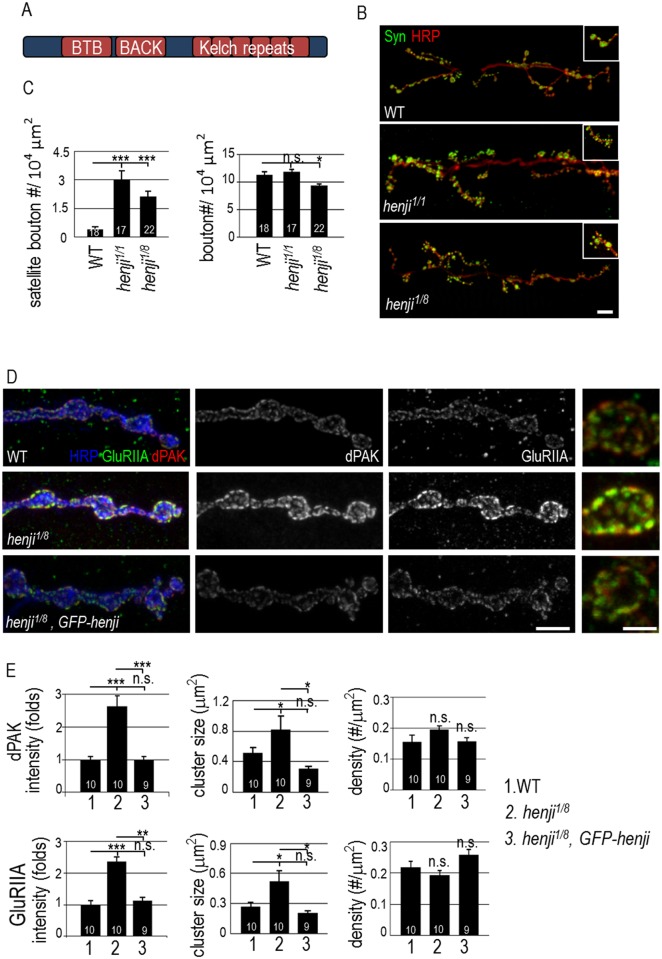
NMJ morphology and dPAK and GluR levels are altered in *henji* mutants. Schematic diagram shows the conserved BTB, BACK and Kelch repeats domains of Henji. (B) Immunostaining of third-instar larval NMJs of *w*^*1118*^ (WT), *henji*^*1*^*/henji*^*1*^ (*henji*^*1/1*^) and *henji*^*1*^*/henji*^*8*^ (*henji*^*1/8*^) with anti-HRP and anti-Synapsin (Syn) antibodies to reveal the overall NMJ morphology. Insets show enlarged images of normal and satellite boutons in each panel. Scale bar represents 20 μm. (C) Bar graphs show mean numbers (± SEM) for satellite boutons normalized to muscle areas (WT, 0.40 ± 0.13; *henji*^*1/1*^, 3.00 ± 0.48; *henji*^*1/8*^, 2.13 ± 0.29; per 10^4^ μm^2^), and for regular boutons normalized to muscle areas (WT, 11.30 ± 0.62; *henji*^*1*^, 11.86 ± 0.44; *henji*^*1/8*^, 9.35 ± 0.38; per 10^4^ μm^2^). (D) Immunostaining for HRP, dPAK and GluRIIA to show NMJs of WT, *henji*^*1/8*^, and *henji*^*1/8*^ *GFP-henji*. Enlarged images for single boutons are on the right side. Scale bars are 10 μm (left) and 5 μm (right), respectively. (E) Bar graphs show means (± SEM) for dPAK fluorescence intensity normalized to HRP intensity (WT, 1.00 ± 0.09; *henji*^*1/8*^, 2.54 ± 0.32; *henji*^*1/8*^,*GFP-henji*, 1.03 ± 0.09), dPAK cluster size (WT, 0.49 ± 0.09; *henji*^*1/8*^, 0.94 ± 0.20; *henji*^*1/8*^,*GFP-henji*, 0.31 ± 0.03 μm^2^), dPAK punctum density normalized to HRP area (WT, 0.16 ± 0.02; *henji*^*1/8*^, 0.20 ± 0.01; *henji*^*1/8*^,*GFP-henji*, 0.16 ± 0.01; # per μm^2^), GluRIIA fluorescence intensity normalized to HRP intensity (WT, 1.00 ± 0.14; *henji*^*1/8*^, 2.18 ± 0.15; *henji*^*1/8*^,*GFP-henji*, 1.28 ± 0.13), GluRIIA punctum size (WT, 0.29 ± 0.05; *henji*^*1/8*^, 0.59 ± 0.11; *henji*^*1/8*^,*GFP-henji*, 0.20 ± 0.02 μm^2^) and GluRIIA punctum density normalized to HRP area (WT, 0.22 ± 0.02; *henji*^*1/8*^, 0.18 ± 0.01; *henji*^*1/8*^,*GFP-henji*, 0.26 ± 0.02; # per μm^2^). Both dPAK and GluRIIA fluorescence intensities normalized to HRP intensities are set as 1 in WT. Statistical significance by unpaired Student t-test is shown as *, p < 0.05; **, p < 0.01; ***, p < 0.001.

At wild-type (WT) NMJs, each bouton is connected to adjacent boutons through linear or bifurcated branches. However, at *henji* NMJs, multiple smaller boutons emerged from large parental boutons (Fig 1B, insets). These smaller boutons, defined as satellite boutons, usually resulted from more than three buds emanating from a parental bouton [35, 36]. Satellite boutons were rarely found at WT NMJs, but were a prominent feature in all *henji* mutants we had examined, including *henji*^*1/1*^ and *henji*^*1/8*^ (Fig 1B). The number of boutons with normal size, however, was either slightly reduced in *henji*^*1/8*^ or remained normal in *henji*^*1/1*^ (Figs 1C and S2C), suggesting that the formation of satellite boutons is not at the expense of normal boutons.

**Fig 2:**
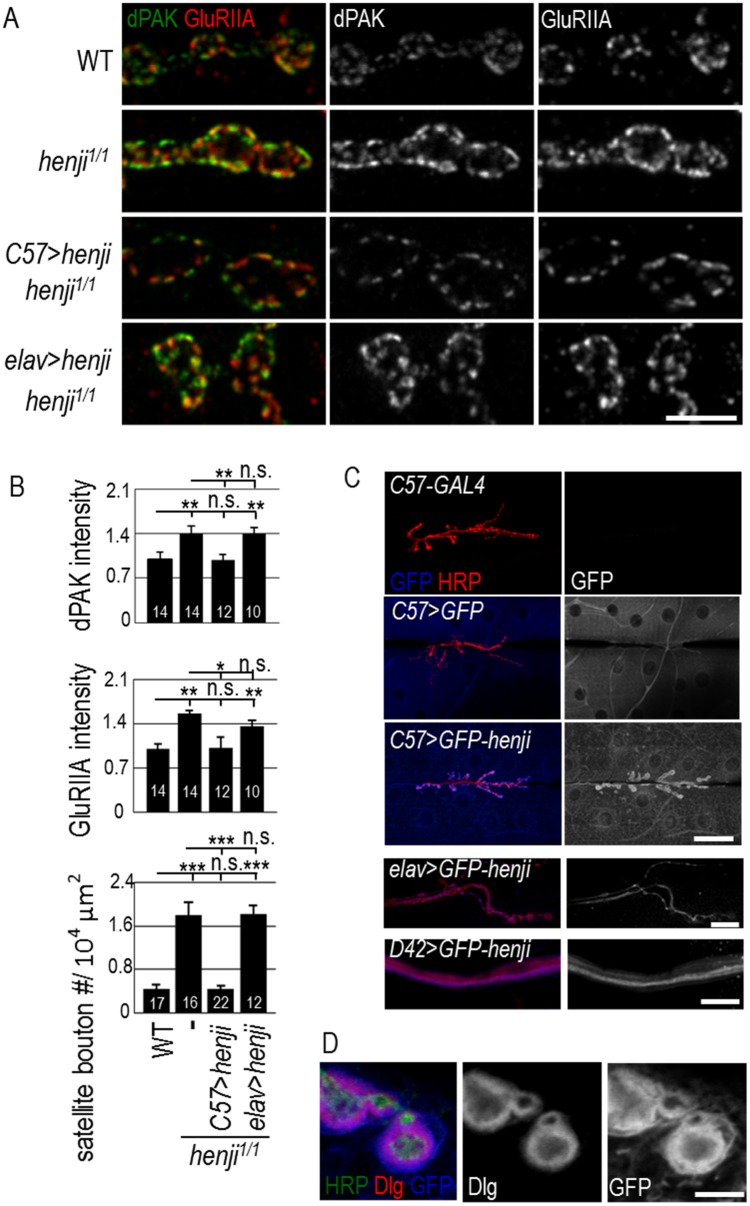
Henji functions in postsynapses. (A) Immunostaining of NMJs for dPAK and GluRIIA in WT, *henji*^*1/1*^, and *UAS-henji* transgene rescue of *henji*^*1/1*^ by muscular *C57-GAL4* (*C57>henji*) or neuronal *elav-GAL4* (*elav>henji*). Scale bar is 5 μm. (B) Bar graphs show fluorescence intensities of dPAK and GluRIIA, and numbers of satellite boutons. dPAK intensity normalized to HRP: WT, 1.00 ± 0.12; *henji*^*1/1*^, 1.41 ± 0.11; *henji*^*1/1*^ *C57>henji*, 0.98 ± 0.09; *henji*^*1/1*^ *elav>henji*, 1.40 ± 0.10. GluRIIA intensity normalized to HRP: WT, 1.00 ± 0.08; *henji*^*1/1*^, 1.57 ± 0.06; *henji*^*1/1*^ *C57>henji*, 1.02 ± 0.17; *henji*^*1/1*^ *elav>henji*, 1.37 ± 0.09. Satellite bouton numbers normalized to muscle area: WT, 0.44 ± 0.08; *henji*^*1/1*^, 1.81 ± 0.24; *henji*^*1/1*^ *C57>henji*, 0.45 ± 0.58; *henji*^*1/1*^ *elav>henji*, 1.82 ± 0.15. Statistical significance by unpaired Student t-test is shown as *, p < 0.05; **, p < 0.01; ***, p < 0.001. (C) Immunostaining for GFP shows Henji localization at NMJs for *C57-GAL4-*driving *GFP-henji* (*C57>GFP-henji*) but not for *C57-GAL4* or *C57-GAL4* driving cytosolic GFP (*C57>GFP*) controls. Scale bar is 50 μm representing top three panels. Presynaptic *elav>GFP-henji* shows NMJ expression with scale bar of 10 μm (fourth panel) and *D42>GFP-henji* shows axonal expression with scale bar of 20 μm (bottom panel). (D) Immunostaining of *C57>GFP-henji* boutons for GFP and Dlg revealed the localization of Henji in and slightly beyond Dlg-positive SSR. Scale bar is 5 μm.

A bouton houses tens of synaptic sites where the presynaptic active zones (AZs) opposes the postsynaptic PSDs. We first examined PSD structure and PSD-localized GluR clusters in *henji* mutants by co-immunostaining with antibodies against PSD-specific dPAK and the GluRIIA subunit (Fig 1D). In WT, dPAK localized as well-separated puncta. In the *henji*^*1/8*^ mutant, the area of individual puncta was expanded and the immunofluorescent intensity was enhanced (Fig 1D). When normalized to co-stained HRP, the dPAK immunofluorescent intensity and punctum size were increased while the density of dPAK puncta was normal, as compared to WT control (Fig 1E, upper bar graphs). As dPAK is required for PSD formation and regulates GluRIIA cluster formation [19], the increase in dPAK levels and patch size suggests a possible enlargement of the PSD that houses GluR clusters. Consistently, GluRIIA immunopositive puncta increased in both intensity and size in the *henji*^*1/8*^ mutant (Fig 1D and 1E). The increases in dPAK and GluRIIA immunointensities were also detected in *henji*^*1/1*^ (Fig 2A and 2B). GluRIIB immunostaining signals in *henji*^*1/8*^, however, showed no significant difference in the intensity to WT control (S2D Fig). These results suggest that Henji regulates the dPAK level at PSDs and specifically confines GluRIIA cluster size. At NMJs lacking *henji*, the increase in GluRIIA but not GluRIIB abundance leads to a shift in the synaptic GluRIIA/GluRIIB ratio.

To examine if *henji* is responsible for the defects observed in *henji* mutants, we generated a genomic rescue construct in which GFP was fused to Henji at the N-terminus, and the *GFP-henji* transgene is driven by the endogenous *henji* promoter. When introduced into *henji*^*1/8*^, *GFP-henji* restored dPAK and GluRIIA to near WT levels (Fig 1D). The intensities of dPAK and GluRIIA immunofluorescent signals showed no significant difference to WT controls (Fig 1E). Thus, the lack of *henji* is the cause for the augmented dPAK and GluRIIA levels at NMJs.

### Postsynaptic Henji regulates bouton morphology and GluRIIA cluster size

The increased PSD size in *henji* mutants prompted us to examine the opposing AZ in presynapses. Bruchpilot (Brp), an essential component of the T-bar structure within AZs [37, 38], was expressed in a normal pattern and intensity at the *henji*^*1/8*^ NMJ (S3A Fig). Each presynaptic Brp punctum matched an enlarged dPAK patch in postsynapses, showing a characteristic pattern between pre- and postsynapses. Compared to control, Brp punctum from the *henji* mutant was unaltered in the intensity, density, and size (S3A Fig, bar graphs). Also, the levels and patterns of the SSR protein Discs large (Dlg), the cell adhesion molecule Fasciculin II (FasII), and the microtubule-associated protein Futsch at *henji* NMJs were indistinguishable to WT controls (S3B Fig).

The specific alterations in dPAK and GluRIIA expressions at *henji* mutant NMJs suggest that Henji functions in postsynapses. To determine the functional site of Henji, we performed a rescue experiment with tissue-specific GAL4 drivers to induce *UAS-henji* expression. As shown, *henji*^*1/1*^ also displayed higher levels of dPAK and GluRIIA in postsynapses (Fig 2A and 2B). When expressed in the *henji*^*1/1*^ postsynapse by muscular *C57-GAL4*, the intensities of both dPAK and GluRIIA puncta at NMJs were suppressed to WT levels (Fig 2A and 2B). In addition, supernumerary satellite boutons in *henji* mutants were also suppressed by postsynaptic expression of *henji* (Fig 2B, bottom panel). In contrast, presynaptic expression of *UAS-henji* using neuronal *elav-GAL4* failed to suppress any of these phenotypes (Fig 2A and 2B). Thus, *henji* is required in postsynapses to regulate postsynaptic dPAK and GluRIIA abundance and presynaptic bouton growth.

With the requirement for *henji* in postsynapses, we examined Henji localization at NMJs. We raised antibodies against Henji, which failed to reveal any specific signal in immunostaining. Also, the *GFP-henji* transgene that was tagged with GFP failed to show any detectable expression level. These results suggest that Henji might be expressed at very low levels. We took advantage of the *GFP-henji* transgene that also includes a UAS for GAL4-induced expression. When muscular *C57-GAL4* was added, GFP-Henji showed localization near the synaptic region (Fig 2C, third row). The postsynaptic-enriched pattern of Henji is specific, as the expression of cytosolic GFP showed diffuse staining in muscle cells without any particular pattern (Fig 2C, second row). Presynaptically expressed GFP-Henji by neuronal GAL4 drivers also displayed diffuse signals in terminal boutons and axonal tracts (Fig 2C, bottom two rows, respectively). To further examine the postsynaptic-enriched expression, co-immunostaining with Dlg was performed. GFP-Henji localized to the SSR and extended slightly outward as compared to Dlg immunostaining (Fig 2D). The postsynaptic localization of Henji suggests a direct mechanism for Henji to regulate the abundance of dPAK and GluRIIA.

### Enlarged postsynaptic quantal size in *henji* mutants

**Fig 3:**
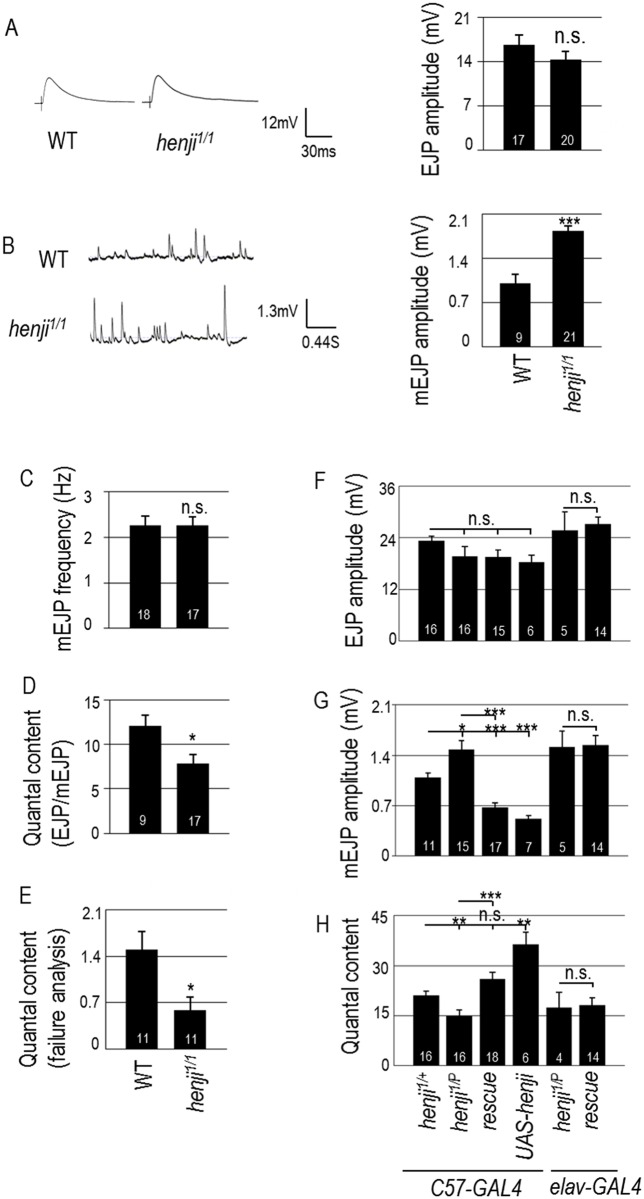
*henji* mutants display larger quantal size while maintaining synaptic homeostasis. (A, B) Representative traces of evoked (A) and spontaneous responses (B) with bar graphs at right for EJP amplitudes of WT, 16.67 ± 1.49 and *henji*^*1/1*^, 14.36 ± 1.81 (A), and mEJP amplitudes of WT, 1.00 ± 0.14; *henji*^*1/1*^, 1.83 ± 0.08 (B). (C-E) Bar graphs show mEJP frequency of WT, 2.26 ± 0.20 and *henji*^*1/1*^, 2.25 ± 0.20 (C), quantal contents calculated as EJP/mEJP, with WT, 12.11 ± 1.24 and *henji*^*1/1*^, 7.82 ± 1.05 (D), and failure analysis at 0.2 mM [Ca^2+^], with WT, 1.50 ± 0.27; *henji*^*1/1*^, 0.59 ± 0.19 (E). (F-H) Bar graphs show pre- or postsynaptic rescue of *henji*^*1/P*^ by *UAS-henji* for (F) EJPs with *C57-GAL4 henji*^*1/+*^, 22.46 ± 1.13; *C57-GAL4 henji*^*1/P*^, 19.96 ± 2.25; *C57>UAS-henji henji*^*1/P*^, 17.08 ± 0.06; *C57>UAS-henji*, 18.29 ± 1.58; *elav-GAL4 henji*^*1/P*^, 25.69 ± 5.01; *elav>UAS-henji*, *henji*^*1/P*^, 27.21 ± 1.56; (G) mEJPs with *C57-GAL4 henji*^*1/+*^, 1.09 ± 0.06; *C57-GAL4 henji*^*1/P*^, 1.48 ± 0.12; *C57>UAS-henji henji*^*1/P*^, 0.68 ± 0.06; *C57>UAS-henji*, 0.52 ± 0.05; *elav-GAL4 henji*^*1/P*^, 1.51 ± 0.22; *elav>UAS-henji*, *henji*^*1/P*^, 1.54 ± 0.13; and (H) quantal contents with *C57-GAL4 henji*^*1/+*^, 21.06 ± 1.26; *C57-GAL4 henji*^*1/P*^, 14.29 ± 1.74; *C57>UAS-henji henji*^*1/P*^, 26.36 ± 2.33; *C57>UAS-henji*, 36.37 ± 3.65; *elav-GAL4 henji*^*1/P*^, 17.51 ± 4.43; *elav>UAS-henji*, *henji*^*1/P*^, 19.53 ± 1.84. Statistical significance by unpaired Student t-test is shown as * for p < 0.05; ** for p < 0.01; *** for p < 0.001; or n.s. for no significance.

Considering the evident GluR compositional shift towards elevated GluRIIA levels in the *henji* mutants, we addressed whether synaptic transmission is also affected by performing electrophysiological recordings. The amplitude of evoked junctional potential (EJP) did not show any defect (Fig 3A). However, the postsynaptic response to spontaneous neurotransmitter release (quantal size), as assessed by measuring the miniature EJP (mEJP) amplitude, was strongly elevated in the *henji*^*1/1*^ mutant (Fig 3B), consistent with an increase in the GluRIIA/GluRIIB ratio. The frequency of mEJP remained normal (Fig 3C). The quantal content, representing the number of effective synaptic vesicles released upon a nerve stimulus, was calculated as the ratio of EJP/mEJP amplitudes. We found that quantal content values decreased significantly in the *henji*^*1/1*^ mutants as compared to WT control (Fig 3D). Similarly, we also detected similar elevation of mEJP and normal EJP in *henji*^*1/P*^, leading to a reduction of the quantal content, as compared to the *henji*^*1/+*^ heterozygous control (Fig 3F–3H, compare first two bars). To further confirm the reduction of the quantal content, failure analysis was performed, which showed decreased release probability in the *henji*^*1/1*^ mutant (Fig 3E). These data suggest that GluRIIA accumulates in the *henji* mutant, causing an elevation in postsynaptic responses. However, homeostatic mechanisms might tune down presynaptic release to reduce the quantal content, thereby maintaining a normal EJP output.

We then tested whether the enhanced mEJP amplitude in the *henji* mutant is caused by the absence of Henji in postsynapses. Muscle expression of *henji* suppressed the mEJP amplitude, both in the *henji*^*1/P*^ mutant and WT background, whereas neuronal expression did not (Fig 3G). As Henji plays a role in suppressing the GluRIIA level in postsynapses (Fig 2A and 2B), the elevation of the GluRIIA level is consistent with the enhancement of mEJP in the *henji* mutant.

### Expansion of synapse size in *henji* mutants

**Fig 4:**
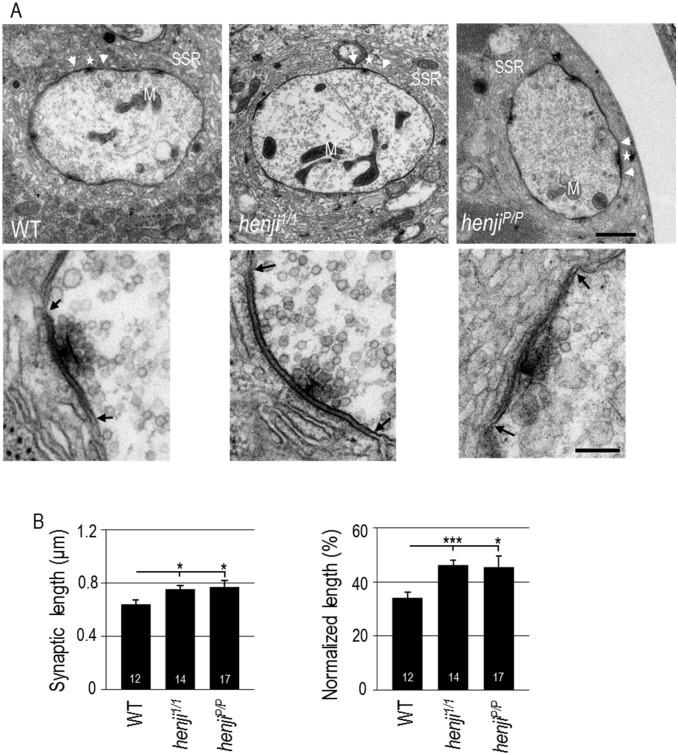
Ultrastructures of enlarged synaptic regions at *henji* NMJs. (A) Electron micrographs show NMJ boutons of WT, *henji*^*1/1*^ and *henji*^*P/P*^ with synaptic regions marked by two arrowheads. T-bars are indicated by stars and mitochondria by M. Enlarged images of synaptic regions within two arrows were shown in bottom panels. Scale bar is 1 μm in the upper panel and 0.2 μm in the lower panel. (B) Synaptic area are expanded in *henji* mutants, with absolute length of dense membrane WT, 0.64 ± 0.03; *henji*^*1/1*^, 0.75 ± 0.03; *henji*^*P/P*^, 0.77 ± 0.05 (μm), and normalized length (to bouton perimeters) in WT, 34.00 ± 2.14; *henji*^*1/1*^, 46.15 ± 1.91; *henji*^*P/P*^, 45.57 ± 4.08 (%). Statistical significance by unpaired Student t-test is shown as * for p < 0.05; ** for p < 0.01; *** for p < 0.001.

The increase of dPAK and GluRIIA patches may be associated with an expansion of the synaptic size in *henji* mutants. To examine this possibility, ultrastructures of boutons were analyzed by TEM. Cross-sections of boutons showed electron-dense membrane regions, representing the matching sites between presynaptic AZ and postsynaptic PSD (Fig 4A, within two arrows). In presynapses, synaptic vesicle-docked T-bars located within AZs, while in postsynapses, membranous SSR enwraps the bouton. We found that the electron-dense membrane region was expanded in *henji* mutants (enlarged panels in Fig 4A). Quantification indicated that the length of the electron-dense membrane region significantly increased in the *henji*^*1/1*^ and *henji*^*P/P*^ mutants (Fig 4B). Moreover, as the bouton perimeter did not differ significantly between *henji* mutants and WT control (S1 Table), the synaptic membrane accounted for a larger proportion of the total membrane region in the lack of *henji* (Fig 4B, right panel). These analyses indicate that the synaptic membrane region is expanded in the *henji* mutants.

### Suppression of *henji* mutant phenotypes by reducing the *dpak* gene dosage

**Fig 5:**
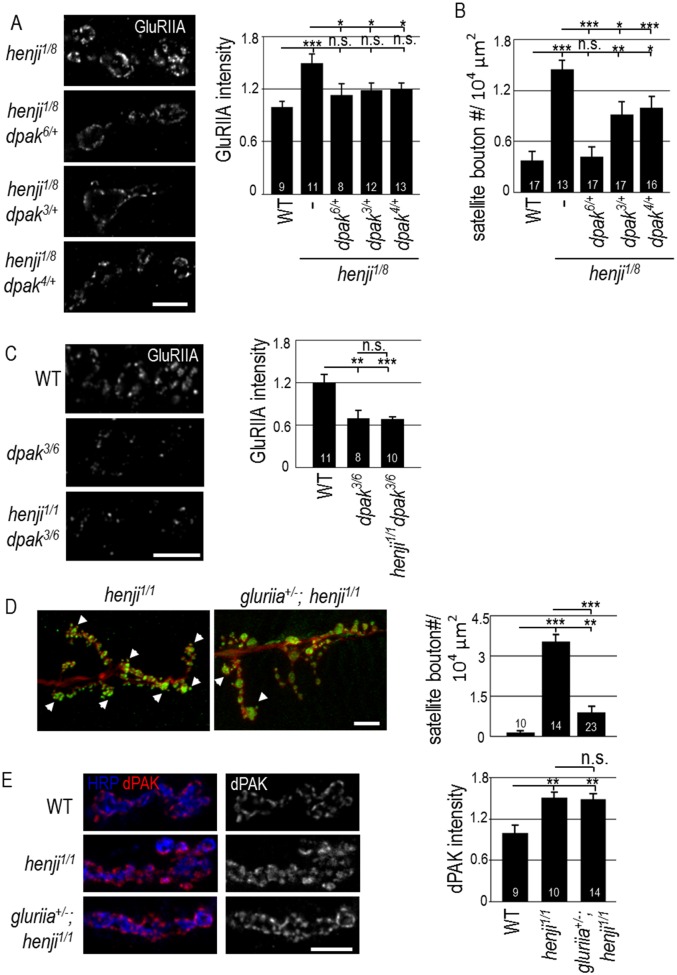
Decreased *dpak* or *gluriia* gene dosages suppress *henji* phenotypes. (A) Immunostaining for GluRIIA in *henji*^*1/8*^ and *henji*^*1/8*^ carrying *dpak* mutations. Scale bar is 5 μm. Bar graph at right shows quantification of GluRIIA intensity normalized to HRP in WT, 1.00 ± 0.06; *henji*^*1/8*^, 1.50 ± 0.11; *dpak*^*6/+*^ *henji*^*1/8*^, 1.13 ± 0.13; *dpak*^*3/+*^ *henji*^*1/8*^, 1.19 ± 0.08; and *dpak*^*4/+*^ *henji*^*1/8*^, 1.20 ± 0.08. (B) Suppression of satellite boutons quantified in WT, 2.82 ± 0.86; *henji*^*1/8*^, 11.31 ± 1.38; *dpak*^*6/+*^ *henji*^*1/8*^, 3.00 ± 0.79; *dpak*^*3/+*^ *henji*^*1/8*^, 7.11 ± 1.17; and *dpak*^*4/+*^ *henji*^*1/8*^, 4.25 ± 1.07. Satellite bouton numbers are normalized to muscle areas (# / 10^4^ μm^2^). (C) Immunostaining of GluRIIA shows suppression of GluRIIA intensity in *dpak henji* double mutants. Bar graph at right shows GluRIIA intensity normalized to HRP intensity. WT, 1.00 ± 0.09; *dpak*^*3/6*^, 0.58 ± 1.00; *dpak*^*3/6*^ *henji*^*1/1*^, 0.57 ± 0.03. (D) Increase of satellite boutons in *henji* is suppressed by removing one copy of *gluriia*. NMJ morphology was revealed by immunostaining for HRP and Syn. Numbers of satellite boutons per 10^4^ μm^2^ are: WT, 1.20 ± 0.49; *henji*^*1/1*^, 30.14 ± 2.58; *gluriia*^*+/-*^ *henji*^*1/1*^, 7.17 ± 1.70. Scale bar is 10 μm. Some satellite boutons are indicated by arrowheads. (E) *gluriia* fails to suppress the elevation of dPAK levels in *henji*, with WT, 1.00 ± 0.11; *henji*^*1/1*^, 1.50 ± 0.09; and *gluriia*^*+/-*^ *henji*^*1/1*^, 1.49 ± 0.08. dPAK intensity is normalized to HRP intensity. Scale bar is 5 μm. Statistical significance by unpaired Student t-test is shown as * for p < 0.05; ** for p < 0.01; *** for p < 0.001, and n.s. for no significance.

Given the elevation of synaptic dPAK levels in the *henji* mutant, we tested whether reducing the *dpak* gene dosage could have an effect on *henji* mutant phenotypes. When the *dpak*^*6*^ null allele was introduced into the *henji*^*1/8*^ mutant background, GluRIIA abundance was suppressed (Fig 5A). Similarly, both kinase-dead *dpak*^*3*^ and Dock-interaction-disrupted *dpak*^*4*^ alleles [39] also suppressed GluRIIA abundance in the *henji*^*1/8*^ mutant, suggesting that these functional domains are critical for dPAK to regulate GluRIIA abundance (Fig 5A). Satellite boutons in the *henji*^*1/8*^ mutant were also suppressed by *dpak*^*6*^ and, to a lesser extent, *dpak*^*3*^ and *dpak*^*4*^ (Fig 5B). In removing one copy of the *dpak*^*6*^ null allele in the *henji*^*1/8*^ mutant, dPAK was indeed reduced to near the WT level (S4 Fig), consistent with the idea that the reduction of the dPAK level is able to suppress *henji* phenotypes. These genetic suppressions suggest that the upregulated dPAK level contributes to GluRIIA accumulation and abnormal bouton morphology in the *henji* mutant.

It has been shown that dPAK regulates synaptic GluRIIA abundance; in the *dpak* mutant, the GluRIIA level was reduced at the PSD [19]. We then examined the epistatic relationship between *henji* and *dpak* mutants. In *dapk*^*3/6*^ larvae that survived to late larval stages, the GluRIIA level was greatly reduced (Fig 5C), consistent with the previous report [19]. In contrast, the GluRIIA level was enhanced in the *henji*^*1/1*^ mutant (Fig 2A). In the *henji*^*1/1*^ *dapk*^*3/6*^ double mutant, GluRIIA was reduced to the level similar to that of the *dapk*^*3/6*^ single mutant (Fig 5C). Thus, in the absence of *dpak*, the GluRIIA level fails to be upregulated in the *henji* mutant, suggesting that *dpak* functions downstream of or in parallel to *henji*.

With the upregulated GluRIIA level in the *henji* mutant, we also examined any suppression effect by reducing GluRIIA in a *henji* mutant background. Introducing one copy of a *gluriia* deletion allele in the *henji* mutant strongly suppressed the satellite bouton phenotype (Fig 5D, arrowheads). However, dPAK abundance was not suppressed by reducing a *gluriia* gene dosage (Fig 5E). Taken together, these data support a model whereby Henji restricts postsynaptic GluRIIA abundance by downregulating the dPAK levels. In *henji* mutants, accumulated GluRIIA induces abnormal bouton growth in the retrograde direction, resulting in satellite bouton morphology.

### Kelch repeats of Henji in suppression of dPAK and GluRIIA

**Fig 6:**
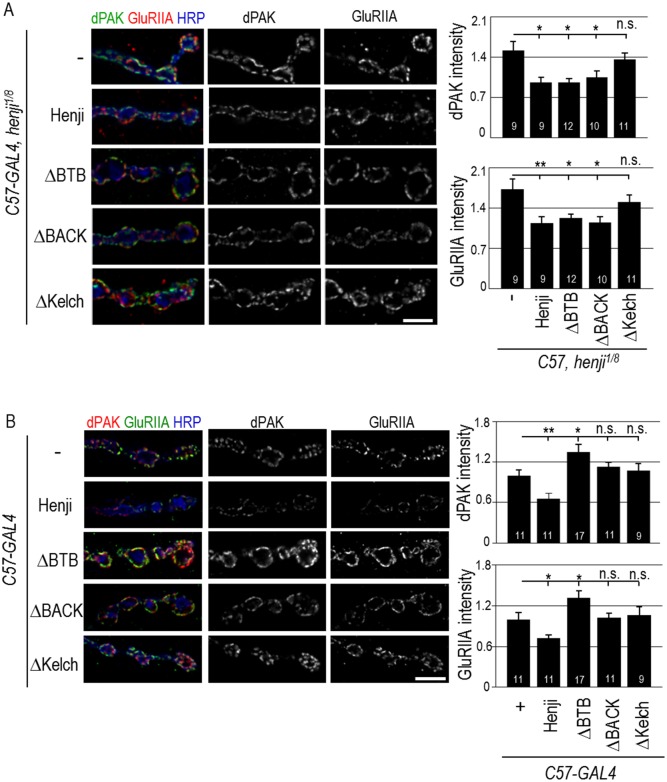
Kelch repeats of Henji suppresses dPAK and GluRIIA elevations in *henji* mutants. (A) GFP-Henji fusion protein and GFP-fused truncations ΔBTB, ΔBACK, and ΔKelch were expressed in muscles by *C57-GAL4* to test the suppression of elevated dPAK and GluRIIA levels at the *henji*^*1/8*^ NMJs that were co-stained with HRP. Scale bar is 5 μm. GluRIIA and dPAK levels were quantified and normalized to HRP. *C57-GAL4 henji*^*1/8*^, 1.73 ± 0.18 and 1.52 ± 0.15; *C57>Henji henji*^*1/8*^, 1.14 ± 0.11 and 0.96 ± 0.09; *C57>ΔBTB henji*^*1/8*^, 1.23 ± 0.07 and 0.96 ± 0.07; *C57>ΔBACK henji*^*1/8*^, 1.15 ± 0.11 and 1.06 ± 0.10; and *C57>ΔKelch henji*^*1/8*^, 1.50 ± 0.13 and 1.36 ± 0.11. (B) Overexpression of full-length Henji, ΔBTB, ΔBACK, and ΔKelch by *C57-GAL4* in muscles to examine their effects on dPAK and GluRIIA postsynaptic levels. dPAK and GluRIIA intensities were quantified and normalized to HRP intensity. *C57-GAL4*, 1.00 ± 0.10 and 1.00 ± 0.08; *C57>Henji*, 0.72 ± 0.05 and 0.66 ± 0.07; *C57>ΔBTB*, 1.31 ± 0.10 and 1.35 ± 0.11; *C57>ΔBACK*, 1.03 ± 0.06 and 1.13 ± 0.07; and *C57>ΔKelch*, 1.06 ± 0.13 and 1.08 ± 0.11). Scale bars are 5 μm in (A, B). Statistical significance was tested by unpaired Student t-test was compared to *C57-GAL4 henji*^*1/8*^ (A) or *C57-GAL4* (B) with * for p < 0.05, ** for p < 0.01, *** for p < 0.001, and n.s. for p > 0.05.

To further understand the role of Henji on postsynaptic regulation, we generated N-terminal GFP-tagged deletion constructs *ΔBTB*, *ΔBACK* and *ΔKelch* that truncate one of the three conserved domains of Henji (Fig 1A). These Henji truncations and full-length control were expressed in postsynapses for rescuing *henji*^*1/8*^ mutant phenotypes. As expected, full-length Henji when expressed in postsynapses suppressed the elevated dPAK and GluRIIA in *henji*^*1/8*^ while *ΔKelch* failed to do so, suggesting that the Kelch repeats region is essential for Henji function in postsynapses (Fig 6A). Surprisingly, *ΔBTB* and *ΔBACK* significantly suppressed elevated intensities of both dPAK and GluRIIA in the *henji*^*1/8*^ mutant. These results suggest that both BTB and BACK domains are dispensable for Henji to function in postsynapses. To further address functional domains of Henji in postsynapses, full-length and truncations of Henji were overexpressed in postsynapses, and synaptic abundances of dPAK and GluRIIA were assessed. Significantly, full-length Henji when overexpressed in postsynapses caused reductions in dPAK and GluRIIA levels at NMJs, suggesting that Henji is sufficient to promote downregulation of dPAK and GluRIIA levels (Fig 6B). Unlike the full-length Henji, truncating any of the three domains failed to downregulate dPAK and GluRIIA levels. Instead, ΔBTB produced a dominant-negative effect by inducing dPAK and GluRIIA accumulations while ΔBACK and ΔKelch had no effects (Fig 6B). Therefore, the Kelch repeats seems to be the most critical domain of Henji to regulate the postsynaptic abundance of dPAK and GluRIIA.

### Kelch repeats of Henji in postsynaptic localization and dPAK interaction

**Fig 7:**
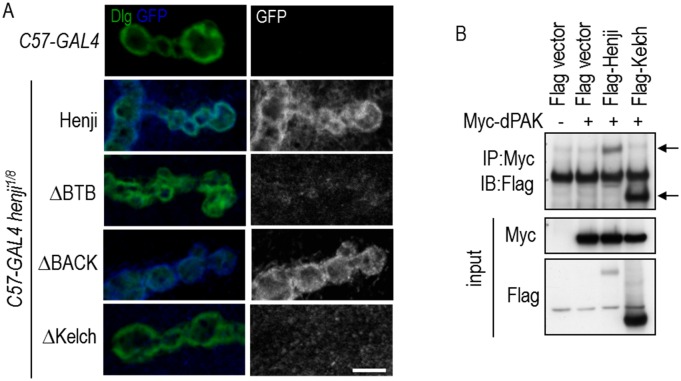
The Kelch repeats in postsynaptic localization and dPAK interaction. (A) Postsynaptic localizations of full-length Henji, ΔBTB, ΔBACK, and ΔKelch expressed by *C57-GAL4* in the *henji*^*1/8*^ mutants were revealed by costaining for Dlg and GFP. Scale bar is 5 μm. (B) Immunoprecipitation of Myc-dPAK complexes (IP) from transfected S2 cells were assayed for the presence of Flag-tagged full-length Henji and the Kelch repeats domain (indicated by arrows) by Western blot (IB). Inputs of dPAK and Henji proteins are at bottom two panels.

We then examined the domain requirement for Henji in postsynaptic localization. Full-length and truncated Henji transgenes were expressed in muscles of the *henji*^*1/8*^ mutant, and the protein localization was detected by GFP immunostaining. By co-staining with Dlg, full-length Henji localized to postsynaptic SSR (Fig 7A). Similarly, absence of the BACK domain (*ΔBACK*) still retained proper synaptic localization of Henji. Lacking the BTB domain also retained some localization signals at the postsynapses. Finally, lacking the Kelch repeats (*ΔKelch*) completely abolished Henji postsynaptic localization (Fig 7A). These analyses suggest that the Kelch repeats region are essential for proper postsynaptic localization of Henji.

Considering the role of Kelch repeats in Henji postsynaptic function and localization, we tested whether Kelch repeats and dPAK interact physically. Indeed, both Flag-tagged full-length and Kelch repeats of Henji co-immunoprecipitated with Myc-tagged dPAK, providing evidence of physical interactions between Henji and dPAK (Fig 7B, indicated by arrows). Taken together, these results indicate that the Kelch repeats that bind to dPAK are required for Henji localization and function to control dPAK and GluRIIA postsynaptic abundances.

### Henji regulates dPAK activity to alter GluRIIA abundance

**Fig 8:**
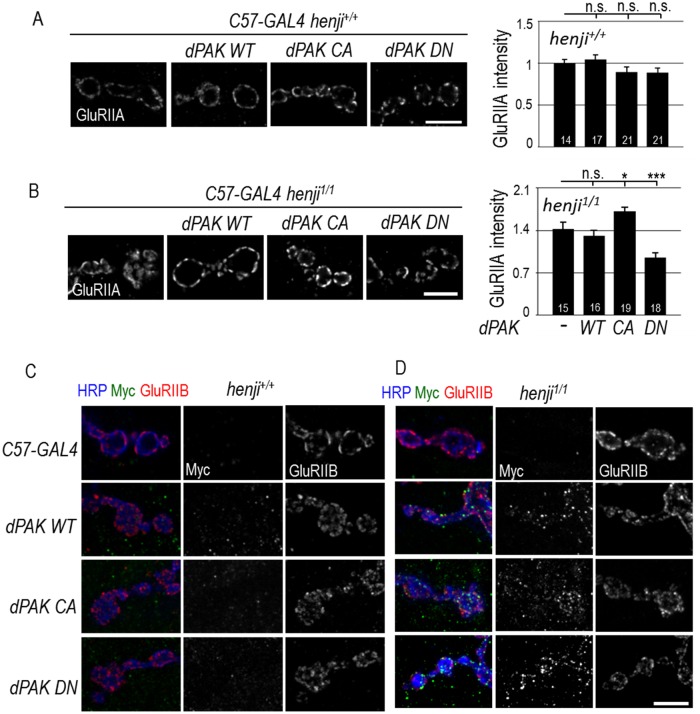
Henji suppresses dPAK activation and localization. (A, B) Overexpression of *dPAK* of *WT*, *CA* and *DN* transgenes in *henji*^*+/+*^ (A) and *henji*^*1/1*^ (B) to assess the effects on GluRIIA abundance. Quantifications of GluRIIA intensities normalized to co-stained HRP intensity for dPAK overexpression in *henji*^*+/+*^ and *henji*^*1/1*^, respectively, are shown in bar graphs (right panels): *C57-GAL4*, 1.00 ± 0.04 and 1.43 ± 0.10; *C57> dPAK WT*, 1.04 ± 0.06 and 1.31 ± 0.08; *C57> dPAK CA*, 0.89 ± 0.06 and 1.72 ± 0.10; *C57> dPAK DN*, 0.89 ± 0.06 and 0.95 ± 0.06. No significant differences could be detected among all transgenes for *henji*^*+/+*^ in comparison to *C57-GAL4* (A, right panel). Statistical significance by unpaired Student t-test is shown as * for p < 0.05 and *** for p < 0.001 when compared to *C57-GAL4 henji*^*1/1*^ (B, right panel). (C, D) Immunostaining of NMJs for HRP, Myc and GluRIIB show localization of Myc-tagged dPAK proteins. GluRIIB serves as the PSD marker. Transgenes of WT, CA, and DN were overexpressed in muscles by *C57-GAL4* in *henji*^*+/+*^ (C) and *henji*^*1/1*^ (D). Scale bars are 5 μm.

While dPAK is required for postsynaptic localization of GluRIIA, how dPAK functions to regulate GluRIIA abundance still remains unknown [19]. A constitutive-active form of dPAK that is membrane-tethered failed to increase GluRIIA abundance at PSDs, hinting another layer of regulation on dPAK. We tested whether Henji plays the critical role on limiting dPAK activity in GluRIIA regulation. To investigate this possibility, we generated Myc-tagged dPAK of WT, constitutive-active (CA), or dominant-negative (DN). The CA form contained a phospho-mimic T583E point mutation at the first autophosphorylation residue [40–42]. The DN form contained three point mutations, with H91L and H94L disrupting binding and activation by Cdc42 and Rac, and K459R eliminating kinase activity. Western blot analysis showed that, when driven by *Tub-GAL4*, WT, CA, and DN were expressed at similar levels (S5 Fig). All three transgenes were overexpressed postsynaptically and GluRIIA abundance was quantified. We found that, as previously reported [19], overexpression of dPAK failed to alter GluRIIA abundance regardless of the activation status (Fig 8A).

To examine whether Henji confers the extra layer of regulation on dPAK activation, dPAK transgenes were overexpressed postsynaptically in the *henji*^*1/1*^ mutant background. Overexpression of dPAK WT did not alter the already enhanced GluRIIA level in the *henji*^*1/1*^ mutant, which may reflect a constraint in dPAK activation such as the requirement of CDC42/Rac1 in dPAK activation [40–42]. Interestingly, overexpression of the CA form further enhanced GluRIIA synaptic intensity in the *henji*^*1/1*^ mutants (Fig 8B), a phenotype that was not detected when dPAK CA was overexpressed in the *henji*^*+/+*^ background (Fig 8A). Also, dPAK DN suppressed GluRIIA intensity in the *henji*^*1/1*^ mutant, a phenotype that was not detected in the *henji*^*+/+*^ background, either (Fig 8A and 8B). Taken together, we propose that Henji limits the action of dPAK to regulate GluRIIA abundance by adding another layer of regulation to conventional phosphorylation-mediated dPAK activation.

Finally, we examined the localization of dPAK by Myc immunostaining. Overexpressed dPAK proteins of WT, CA and DN forms showed dispersed weak puncta in muscle cells without forming specific patterns (Fig 8C). When overexpressed in the absence of *henji* activity, Myc-positive puncta became brighter and accumulated around the synaptic region. Some of the puncta were co-labeled with GluRIIB, representing specific accumulation at PSDs (Fig 8D). Taken together, these analyses suggest that Henji functions to limit dPAK from localization at postsynaptic sites, which is important for modulating GluRIIA abundance.

## Discussion

**Fig 9:**
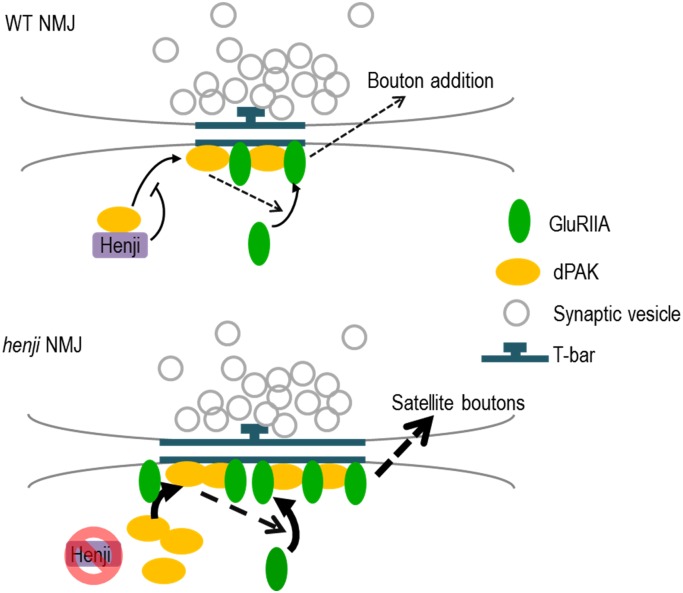
Schematic diagram shows model for Henji function at the NMJ. At WT NMJs, Henji localizes at the SSR and binds with dPAK, preventing dPAK from localizing to the PSDs. With dPAK synaptic levels under control, the rate of GluRIIA clustering into PSDs is limited as dPAK is unavailable. At the *henji* mutant NMJ, unbound dPAK is activated and accumulates at PSDs to promote GluRIIA clustering and accumulation. Excess GluRIIA enhances presynaptic bouton addition, causing satellite boutons to form. Dashed arrows indicate unknown mechanisms. Curved arrows indicate localization of molecules into PSDs.

Here, we show that Henji functions at the postsynapse to regulate synaptic development and function at the NMJ. The PSD area is expanded and GluRIIA clusters abnormally accumulate at the PSD. We provide genetic evidences to support that the elevation of GluRIIA synaptic abundance is at least partially caused by a corresponding accumulation of dPAK in *henji* mutants. We also show that Henji is sufficient to downregulate dPAK and GluRIIA levels and the Kelch repeats of Henji play the most critical role in this process. Henji tightly gates dPAK in regulating GluRIIA abundance, as dPAK enhances GluRIIA cluster abundance only when Henji is absent. Therefore, we have identified a specific negative regulation of dPAK at the postsynaptic sites that contributes to the PSD formation and GluR cluster formation at the NMJ (Fig 9).

### dPAK levels are tightly regulated at the synapse

PAK proteins transduce various signaling activities to impinge on cytoskeleton dynamics. Through kinase activity-dependent and -independent mechanisms, PAK regulates not only actin- and microtubule-based cytoskeletal rearrangement but also the activity of motors acting on these cytoskeletal tracks [43–46]. In mammalian systems, PAKs participate in many synaptic events including dendrite morphogenesis [47, 48], neurotransmitter receptor trafficking [49, 50], synaptic strength modulation [51], and activity-dependent plasticity [52]. Pathologically, PAK dysregulation also contributes to serious neurodegenerative diseases [53], such as Alzheimer disease [54, 55], Huntington’s disease [56, 57] and X-linked mental retardation [58–60].

At *Drosophila* NMJs, dPAK has divergent functions; loss of *dpak* causes a dramatic reduction in both Dlg and GluRIIA synaptic abundance [19], but the underlying molecular mechanisms have not been revealed. Our data show that Henji functions to restrict GluRIIA clustering but has no effect on Dlg levels (S3B Fig), suggesting that Henji regulates one aspect of dPAK activities, probably via the SH2/SH3 adaptor protein Dock [19]. Alternatively, Henji may function to limit dPAK protein levels locally near the postsynaptic region, rendering its influence on GluRIIA clustering, while dPAK that regulates Dlg may localize outside of the Henji-enriched region. Supporting this idea, Henji is specifically enriched around the SSR region instead of dispersed throughout the muscle cytosol (Fig 2D). Moreover, ectopic Myc-dPAK localized at the postsynapse only when *henji* was mutated (Fig 8C and 8D), indicating that Henji regulates dPAK postsynaptic localization.

The interaction with Rac, Cdc42, or both triggers autophosphorylation and subsequent conformational changes of PAK, resulting in kinase activation. The myristoylated dPAK that has been shown to be active in growth cones [39] failed to enhance GluRIIA abundance at the NMJ [19]. This result shows that dPAK is necessary to regulate GluRIIA synaptic abundance, but is itself tightly regulated at the synaptic protein level or the kinase activity. Indeed, we provide evidence to show specific negative regulation of dPAK by Henji; overexpression of dPAK CA that could not enhance GluRIIA abundance in WT larvae further increased the already enhanced GluRIIA levels in the *henji* mutant (Fig 8A and 8B). Similar to the CA form, the DN form also showed no effect on GluRIIA when simply overexpressed in the WT background, but exhibited strong suppression of GluRIIA in the *henji* mutant background (Fig 8A and 8B). Thus, regardless of the possible conformational differences between the CA and DN forms, Henji appears to confer a constitutive negative regulation of dPAK at postsynapses, suggesting a tight control that could be at subcellular localization. In contrast to CA and DN forms, activation of dPAK requires binding to Rac1 and Cdc42, and subsequent protein phosphorylation. This additional layer of regulation may serve as a limiting factor rendering dPAK WT from recruiting GluRIIA to PSDs regardless in WT or *henji* mutant background.

The structural feature suggests that Henji could function as a conventional substrate receptor of the Cul3-based E3 ligase complex. At *Drosophila* wing discs, Dbo functions as a Cul3-based E3 ligase to promote Dishevelled (Dsh) downregulation [61]. Similar to the *henji* alleles, we confirmed that the *dbo [Δ25*.*1]* allele and *dbo RNAi* were competent to induce dPAK and GluRIIA accumulation at the postsynapse (S6A Fig). In our immunoprecipitation experiment, we detected Henji and dPAK in the same complex (Fig 7B, lane 3), and dPAK also forms a complex with the C-terminal substrate-binding Kelch-repeats region (lane 4). However, we did not detect any notable or consistent increase in Henji-dependent dPAK poly-ubiquitination in both S2 cells and larval extracts. Also, the Cul3-binding BTB domain of Henji seems dispensable in the suppression of dPAK levels in *henji* mutants (Fig 6A). Importantly, Cul3 knockdown in muscle cells failed to cause any accumulation of GluRIIA and dPAK at the NMJ (S6A Fig). Sensitive genetic interaction between *henji* and *Cul3* failed to induced dPAK and GluRIIA accumulation (S6B Fig). Dbo functions together with another BTB-Kelch protein Kelch (Kel) to downregulate Dsh [61]. However, Kel negatively regulates GluRIIA levels without affecting dPAK localization at the postsynaptic site (S6C Fig). This data argues that Kel functions in a distinct pathway to Henji in postsynaptic regulation of GluRIIA. Taken together, we found no direct evidence to support that dPAK is downregulated by Henji through ubiquitination-dependent degradation. Alternately, Henji could bind dPAK near the postsynaptic region and this interaction may block the recruitment or localization of dPAK onto postsynaptic sites (Fig 9). Under this model, dPAK is less restricted and has a higher propensity to localize at postsynaptic sites in the absence of Henji, resulting in synaptic accumulation of dPAK and GluRIIA expansions.

### Henji limits dPAK levels to restrict GluRIIA synaptic abundance

As many synaptic events require rapid responses, local regulation of protein levels becomes crucial in synapses. To achieve accurate modulation, certain synaptic proteins should be selectively controlled under different developmental or environmental contexts. Indeed, emerging evidence shows that various aspects of synapse formation and function are under the control of the ubiquitin proteasome system (UPS), including synapse formation [62, 63], morphogenesis [64], synaptic pruning and elimination [65, 66], neurotransmission [67–69], and activity-dependent plasticity [21, 70]. In particular, the membrane abundance of postsynaptic GluR that modulates synaptic function can be regulated by components of the UPS. When *Apc2*, the gene encoding *Drosophila* APC/C E3 ligase, is mutated, GluRIIA shows excess accumulation but the molecular mechanism was not elucidated [71]. Similarly, loss of the substrate adaptor BTB-Kelch protein KEL-8 in *C*. *elegans* also results in the stabilization of GLR-1-ubiquitin conjugates [72]. However, no evidence shows direct ubiquitination and degradation of GLR-1 by KEL-8. Also, absence of the LIN-23-APC/C complex in *C*. *elegans* affects GLR-1 abundance at postsynaptic sites without altering the level of ubiquitinated GLR-1. Therefore, GLR-1 receptor endocytosis and recycling or ubiquitination and degradation of GLR-1-associated scaffold proteins are proposed to be the underlying mechanism for E3 ligase regulation [23, 67]. In mammals, endocytosis of AMPAR can be influenced by poly-ubiquitination and degradation of the prominent postsynaptic scaffold protein PSD-95 [21, 73].

In this study, we describe a novel regulation by the BTB-Kelch protein Henji on synaptic GluRIIA levels. By limiting GluRIIA synaptic levels, Henji modulates the postsynaptic output in response to presynaptic glutamate release. In the absence of Henji, quantal size is elevated (Fig 3B and 3G), coinciding with an increase in the postsynaptic GluRIIA/GluRIIB ratio (Figs 1D and 1E and S2D). In a previous study, increases in the GluRIIA/GluRIIB ratio by overexpressing a GluRIIA transgene in the muscle or by reducing the gene copy of *gluriib* promote NMJ growth, but co-expression of both GluRIIA and GluRIIB did not alter the bouton number [74]. Combined with our findings, those data provide a link between an increased GluRIIA-mediated postsynaptic response and bouton addition at NMJs. However, satellite boutons were not detected following GluRIIA overexpression [74]. One possibility is that satellite boutons are considered as immature boutons [36, 75] and their appearance may indicate the tendency for NMJ expansion, as in the case of excess BMP signaling [75]. Failure to become mature boutons may be caused by the lack of cooperation with other factors such as components of the presynaptic endocytic pathway [76], actin cytoskeleton rearrangement [77–79] or neuronal activity [36]. We found no significant alterations in endocytosis and the BMP pathway in the *henji* mutant (S7 Fig). Nevertheless, we cannot rule out that Henji may modulate other presynaptic events that are defective in *henji* mutants to interfere with bouton maturation (S1 Table).

## Materials and Methods

### Fly stocks

*w*^*1118*^ was used as the WT control and to backcross all *henji* alleles described in this study. Flies of all genotypes were reared at 25°C for experiments. We performed P-element-mediated imprecise excision to generate *henji*^*1*^ and *henji*^*8*^ alleles (S2A Fig). Plasmids of *UAS-Flag-henji* (full-length), *UAS-Flag-Kelch* (533–623 a.a.), *UAS-GFP-henji* (full-length), *UAS-GFP-ΔBTB* (delete 1–167 a.a.), *UAS-GFP-ΔBACK* (delete 168–276 a.a.), *UAS-GFP-ΔKelch* (delete 305–623 a.a.), *UAS-Myc-dpak WT* (full-length), *UAS-Myc-dpak CA* (point mutation T583E) and *UAS-Myc-dpak DN* (triple mutations H91L, H94L and K459R) were constructed using the Gateway System into the pUAST vector (Invitrogen and *Drosophila* Genomics Resource Center, DGRC). The genomic rescue transgene *GFP-henji* was constructed by fusing *GFP* to the ATG codon of *henji* cDNA and driven by the putative promoter region containing the genomic sequence between *CG6169* ATG and *henji* ATG. *C57-GAL4*, *elav-GAL4*, *kel*^*DE1*^, and *dpak*^*6*^ were from Bloomington *Drosophila* Stock Center (BDSC). RNAi lines for *Cul3* (109415) and *gbb* (5562R) [80] were from Vienna Drosophila RNAi Center (VDRC) and National Institute of Genetics (NIG), respectively. Mutant strains that have been described are *dpak*^*3*^ and *dpak*^*4*^ [39], *dbo[Δ25*.*1]* and *dbo* RNAi [61], and *GluRIIA-GFP* rescuing *gluriia* and *gluriib* double mutants [81].

### Immunostaining and image acquisition and processing

Primary antibodies used were: mouse anti-Dlg (4F3, 1:100, Developmental Studies Hybridoma Bank, DSHB), mouse anti-GluRIIA (1:100, DSHB), rabbit anti-dPAK (1:1000)[82], rabbit anti-GluRIIB (1:1000)[14], mouse anti-Brp (1:100, DHSB), mouse anti-FasII (1:100, DSHB), mouse anti-Futsch (1:100, DSHB), chicken anti-GFP (1:100; Abcam Co.), mouse anti-Myc (9E10, 1:100, Santa Cruz Co.), rabbit anti-pMAD (1:250)[83] and rabbit or goat anti-HRP conjugated FITC, TRITC and Cy5 (Jackson ImmunoResearch Laboratories). Secondary antibodies used were anti-rabbit or -mouse Cy3 and Cy5 (Jackson ImmunoResearch Laboratories). Muscles, though not shown in figures, were revealed by staining with FITC-conjugated phalloidin (1:1000; Sigma Co.).

A2 to A6 segments of NMJ4s and A3 segments of NMJ6/7s of wandering third instar larvae were analyzed. Larvae were dissected in cold calcium-free HL3 saline (70 mM NaCl, 5 mM KCl, 20 mM MgCl_2_, 10 mM NaHCO_3_, 5 mM trehalose, 115 mM sucrose, and 5 mM HEPES, pH 7.2) and larval fillets were fixed in 4% paraformaldehyde for 20 min and washed in PBT (0.03% triton-X-100) for 10 min three times. For GluRIIA and GluRIIB staining, larval fillets were fixed in Bouin’s fixative (Sigma Co.) for 5 min. Fixed fillets were incubated with primary antibodies overnight at 4°C, washed in PBT three times, and incubated with secondary antibodies for 2 hr at room temperature. Larval fillets were mounted in solution containing PBS with 87.5% glycerol and 0.22 M 1, 4-diaza-byciclo (2.2.2) octane (Dabco, Sigma Co.). Images were acquired via LSM 510 confocal microscopy (Carl Zeiss) using 40x water and 100x oil objectives. Images were processed by LSM5 image examiner (Carl Zeiss) and Adobe Photoshop Creative Suite, and further quantified by Image J for immunofluorescence intensities, punctum densities and cluster sizes. For quantification, Z-section images were projected for further processing. Immuno-positive regions were defined by using Image J in which threshold setting was used to eliminate background noise. Intensity of each synaptic protein was normalized to corresponding HRP intensity. Satellite bouton numbers were normalized to corresponding muscle areas. For image presentation, immunostaining images presented in figures represent single sections except the Futsch images in S3B Fig were from projection of Z sections. Unpaired Student t-test is used in calculating statistical significance.

### Immunoprecipitation and western blot

*Drosophila* S2 cells were maintained in Schneider's medium (Thermo Fisher Scientific) at 25°C. S2 cells (5 x 10^6^ cells in each 10 cm dish) were transfected with 1 μg DNA of individual constructs using Cellfectin (Invitrogen). S2 cells were collected and homogenized in RIPA lysis buffer (20 mM Tris-HCl, pH8.0, 150 mM NaCl, 5 mM EDTA, 1% Triton-X-100, 2 mM Na_3_VO_4_, 50 mM NaF and 1 mM PMSF, supplemented with protease inhibitor cocktail (Roche). Protein concentrations were calculated with the aid of protein assay (Bio-Rad Laboratories). For immunoprecipitation, Myc-tagged dPAK was co-transfected with either Flag-tagged Henji or the Kelch repeats domain of Henji into S2 cells. Cell lysates were incubated with beads coated with anti-Myc (9E10, Santa Cruz) and the immunoprecipitates were blotted with mouse anti-Flag antibody (1:1000, Sigma Co.). Antibodies used for immunoblotting were anti-dPAK (1:5000), anti-Myc (1:1000), and anti-α-tubulin (1:200000, Sigma Co.).

### Transmission electron microscopy

Larval fillets were dissected in cold calcium-free HL3 saline and subsequently fixed overnight in modified Trump’s universal fixative (4% paraformaldehyde, 1% glutaraldehyde in 0.2 M cacodylate buffer, pH 7.2). The PELCO BioWave^®^ laboratory microwave system was used for subsequent steps. Samples were post-fixed with 1% aqueous osmium tetroxide in 0.2 M cacodylate buffer (pH 7.2) under 20 inHg vacuum. After stained with 2% uranyl acetate for 30 min at room temperature, samples were dehydrated in gradually-increasing ethanol concentrations (50%, 70%, 80% and 90%). Later, fillets were infiltrated in Spurr’s resin with gradual increases of concentrations (25%, 50%, 75% and 100%). Ultrathin sections, obtained by ultramicrotome (Leica), were further stained with uranyl acetate and lead citrate. Images were viewed by Tecnai G2 Spirit TWIN (FEI Company, Hillsboro, OR). Electron-dense region was determined by Gatan DigitalMicrograph. A line was drawn along the bouton membrane and spots with lowest intensity were picked and labeled as the boundary of electron-dense region. Bouton parameters were quantified by Image J.

### Electrophysiological recordings

For sample preparation, larvae were dissected with the segmental nerves cut close to the ventral ganglion region in cold modified calcium-free HL3.1 saline (70 mM NaCl, 5 mM KCl, 10 mM MgCl_2_, 10 mM NaHCO_3_, 5 mM trehalose, 115 mM sucrose, 5 mM HEPES, pH 7.2). Samples were then incubated in modified HL3.1 saline containing 0.8 mM CaCl_2_ for stimulation, and recordings were taken at room temperature. The two electrodes for voltage-clamping were filled with 3 M KCl and impaled in muscle 6 of the A3 segment. One microelectrode (15~20 MΩ) monitored the muscle membrane potential while the other (5~8 MΩ) delivered electric currents. 5-8V stimulation was given to stimulate the nerve. The muscle membrane potential was clamped at -60 mV. Without any stimulation on the segmental nerves, mEJPs within 100 sec were recorded. For evoking an EJP, the segmental nerve was stimulated by a suction electrode every 30 sec with pulse duration of 0.1 msec at the voltage two times that of the threshold. For failure analysis EJP is evoked in 0.2 mM [Ca^2+^], the failure rate was calculated by ln(n/N), with n the number of failure events, and N the total number of stimuli [16]. For high-frequency stimulation, the segmental nerve was stimulated at 13.3 Hz for 4 min in 2 mM [Ca^2+^] buffer. Data were digitized by a DigiData 1440 interface (Molecular Devices) at 50 kHz, and weak signals were filtered at 10 kHz, and analyzed by Clampfit10 (Molecular Devices).

## Supporting Information

S1 Fig Phylogenic tree of *Drosophila* BTB proteins. 60 BTB domain-containing proteins in the fly genome are grouped according to second conserved domains and are shaded with different colors: zinc finger in blue, Psq in pink, Ankyrin repeats in green, MATH in purple, and Kelch repeats in yellow. Those with other domains are not shaded with color.
(PDF)

S2 Fig Characterization of *henji* mutant alleles. (A) Schematic diagram shows the locations of *henji* mutant alleles. *P{GT1}Dcp2[BG01766]* and *P{EPg}dbo*^*HP30996*^ were excised to generate genomic deletions 1 and 8, respectively. *deletion1* truncates part of the 5’ UTRs of the neighboring gene *CG6169* and *henji (CG6224)*. *deletion8* truncates a larger part of the 5’UTR of *CG6169* and the putative translation start site of *henji* (shown as ATG). Since these two deletion mutants also truncate parts of *CG6169*, they were complemented with a *CG6169* genomic rescue construct in the mutant flies for phenotypic analysis. After introducing *CG6169-GR*, the early lethality of both deletion lines was rescued, suggesting that lethality resulted from *CG6169* and that *henji* was a non-essential gene. The mutant carrying *deletion1* or *deletion8* and *CG6169-GR* was named as *henji*^*1*^ or *henji*^*8*^, respectively. The third mutant allele carried a *PBac{PB}dbo*^*c04604*^ transposon insertion in the 5’UTR of *henji*, and this allele was named *henji*^*P*^ in this study. (B) RT-PCR reveals the *henji* mRNA levels in different mutant alleles. *henji*^*1*^, reducing *henji* gene transcription, is a strong loss-of-function allele. The hypomorphic allele *henji*^*P*^ shows reduced mRNA levels compared with WT. *henji*^*8*^, with no detectable *henji* mRNA, is considered a null allele. (C) Bar graph (first from left) shows total bouton numbers of both regular and satellite boutons for WT, 90.89 ± 3.69; and *henji*^*1/1*^, 102.00 ± 4.67; *henji*^*1/8*^, 75.86 ± 3.46. The second bar graph shows average muscle areas for WT, 7.84 ± 1.60; *henji*^*1/1*^, 6.95 ± 0.25; and *henji*^*1/8*^, 6.73 ± 0.28. The third bar graph shows bouton size for WT, 23.44 ± 2.34; and *henji*^*1/1*^, 23.56 ± 1.53. The forth bar graph shows NMJ areas for WT, 2121.69 ± 193.22; and *henji*^*1/1*^, 2323.85 ± 237.41. **, p < 0.01 by unpaired Student t-test. Comparisons with no significant differences (p > 0.05) are indicated by n.s. (D) Co-immunostaining of GluRIIA and GluRIIB shows the significant increase in GluRIIA intensity in *henji*^*1/8*^. However, the GluRIIB intensity was not altered. Presynaptic membranes are revealed by HRP labeling. Enlarged images of single boutons are shown. Scale bars are 5 μm. Right bar graph for quantification of GluRIIB intensity that is normalized to HRP (WT, 1.00 ± 0.12; *henji*^*1/8*^, 1.14 ± 0.08). No significance is detected by unpaired Student t-test.
(PDF)

S3 Fig Normal expressions of synaptic proteins in *henji* mutants. (A) Co-staining of dPAK and Brp shows dPAK accumulation in the *henji*^*1/8*^ mutant but Brp remains at normal levels, as compared to heterozygous *henji*^*1/+*^ controls. Enlarged images shows single boutons with matching Brp and dPAK puncta in both genotypes. Lower bar graphs show no significant differences (n.s.) in Brp intensity (normalized to HRP intensity), and Brp punctum density (normalized to HRP area) and size when comparing both genotypes. Brp intensity (*henji*^*1/+*^, 1.00 ± 0.09; *henji*^*1/8*^, 1.00 ± 0.05); punctum density (*henji*^*1/+*^, 1.00 ± 0.14; *henji*^*1/8*^, 0.78 ± 0.09); Brp punctum size (*henji*^*1/+*^, 0.18 ± 0.02; *henji*^*1/8*^, 0.14 ± 0.08). (B) Dlg, Futsch and FasII immunostaining show no significant differences between *henji*^*1/+*^ and the *henji*^*1/8*^. Bar graphs on the right show HRP-normalized intensities with no significant differences (n.s.) detected by unpaired Student t-test. Dlg: *henji*^*1/+*^, 1.00 ± 0.14; *henji*^*1/8*^, 0.76 ± 0.06; FasII: *henji*^*1/+*^, 1.00 ± 0.09; *henji*^*1/8*^, 1.24 ± 0.31; Futsch: *henji*^*1/+*^, 1.00 ± 0.08; *henji*^*1/8*^, 1.07 ± 0.13. Scale bar is 5 μm.
(PDF)

S4 Fig Reduction of dPAK levels in the *henji*^*1/8*^ mutant by different *dpak* alleles. Bar graph shows the synaptic dPAK levels at NMJs that were normalized to co-stained HRP with WT set as 1. WT, 1.00 ± 0.07; *henji*^*1/8*^, 1.75 ± 0.09; *henji*^*1/8*^ *dpak*^*6/+*^, 1.28 ± 0.13; *henji*^*1/8*^ *dpak*^*3/+*^, 1.51 ± 0.10 and *henji*^*1/8*^ *dpak*^*4/+*^, 1.74 ± 0.13. Significance by unpaired Student t-test is shown with * for p < 0.05, ** for p < 0.01, *** for p < 0.001, and n.s. for no significance.
(PDF)

S5 Fig Similar expression levels of Myc-dPAK WT2, CA, and DN. Myc-dPAK WT1, WT2, CA and DN transgenes were overexpressed ubiquitously by *Tub-GAL4*. Adult fly head extracts were collected for Western blot analysis by anti-Myc antibodies. Although WT1 shows a very low expression level, WT2, CA, and DN have similar expression levels. WT2 were used in this study. Immunoblotting of α-Tubulin (α-Tub) serves as a control.
(PDF)

S6 Fig Cul3 is not involved in Henji-mediated dPAK and GluRIIA regulation. (A) Immunostaining of GluRIIA and dPAK in WT, muscle knockdown of *Cul3*, *dbo[Δ25*.*1]* and muscle knockdown of *dbo* [61]. Quantification of GluRIIA and dPAK intensities with normalization to HRP intensities and the WT value is set as 1. GluRIIA: WT, 1.00 ± 0.06; *C57>Cul3 RNAi*, 1.16 ± 0.07; *dbo[Δ25*.*1]*, 1.98 ± 0.11, and *C57>dbo RNAi*, 1.51 ± 0.14. dPAK: WT, 1.00 ± 0.08; *C57>Cul3 RNAi*, 1.02 ± 0.09; *dbo[Δ25*.*1]*, 1.85 ± 0.13 and *C57>dbo RNAi*, 1.59 ± 0.09. (B) Immunostaining shows no elevation of GluRIIA and dPAK levels in double heterozygous *Cul3*^*C7/+*^ *henji*^*8/+*^ mutants as compared to *Cul3*^*C7/+*^. Quantification of GluRIIA and dPAK intensities normalized to HRP intensities. Satellite bouton numbers are normalized to muscle areas. GluRIIA: *Cul3*^*C7/+*^, 1.00 ± 0.16; *Cul3*^*C7/+*^ *henji*^*8/+*^, 0.97 ± 0.12. dPAK: *Cul3*^*C7/+*^, 1.00 ± 0.09; *Cul3*^*C7/+*^ *henji*^*8/+*^, 0.90 ± 0.08. Satellite boutons: *Cul3*^*C7/+*^, 0.10 ± 0.07; *Cul3*^*C7/+*^, *henji*^*8/+*^, 0.12 ± 0.07. (C) Co-immunostaining of dPAK and GluRIIA in WT and *kel*^*DE1*^ homozygotes. dPAK and GluRIIA intensities were quantified and normalized to HRP intensity. dPAK: WT, 1.00 ± 0.13; *kel*^*DE1*^, 0.84 ± 0.06, and GluRIIA: WT, 1.00 ± 0.13; *kel*^*DE1*^, 1.46 ± 0.15. Significance by unpaired Student t-test is shown with * for p < 0.05, ** for p < 0.01, *** for p < 0.001, and n.s. for no significance. Scale bars are 5μm.
(PDF)

S7 Fig Normal endocytosis and BMP signaling in *henji* mutants. (A) FM1-43 dye uptake is used as an indicator for endocytosis in boutons of WT and *henji*^*1/1*^. Intensity of FM1-43 was quantified and no significant difference was detected between WT and *henji*^*1/1*^. FM1-43 intensity inside boutons: WT, 13.51 ± 3.23; *henji*^*1/1*^, 14.78 ± 2.87. Scale bar is 10 μm. (B) High-frequency stimulation in 2 mM [Ca^2+^]. No significant defect was found in *henji* mutant. (C) Immunostaining of pMAD in the *henji*^*1/+*^ heterozygous control and the *henji*^*1/8*^ mutant. The pMAD intensities were quantified and normalized to HRP intensities. *henji*^*1/+*^, 7.41 ± 1.01; *henji*^*1/8*^, 7.23 ± 0.55. No significance (n.s.) was detected between two genotypes. Scale bar is 5 μm. (D) Knockdown *gbb* in the postsynaptic muscle cells has no suppressing effect on satellite boutons in the *henji*^*1/8*^ mutant. Satellite bouton numbers were normalized to muscle areas. WT, 0.27 ± 0.09; *henji*^*1/8*^, 1.57 ± 0.21; *C57> gbb RNAi henji*^*1/8*^, 1.60 ± 0.24. *** for p < 0.001 and n.s. for no significance by unpaired Student t-test.
(PDF)

S1 Table Ultrastructural parameters from TEM analysis. (DOCX)
