# Supplementary figures and images for: Dbo/Henji Modulates Synaptic dPAK to Gate Glutamate Receptor Abundance and Postsynaptic Response
Source: PLoS Genet. 2016 Oct 13;12(10):e1006362. doi: 10.1371/journal.pgen.1006362 (PMC5065118; doi:10.1371/journal.pgen.1006362)

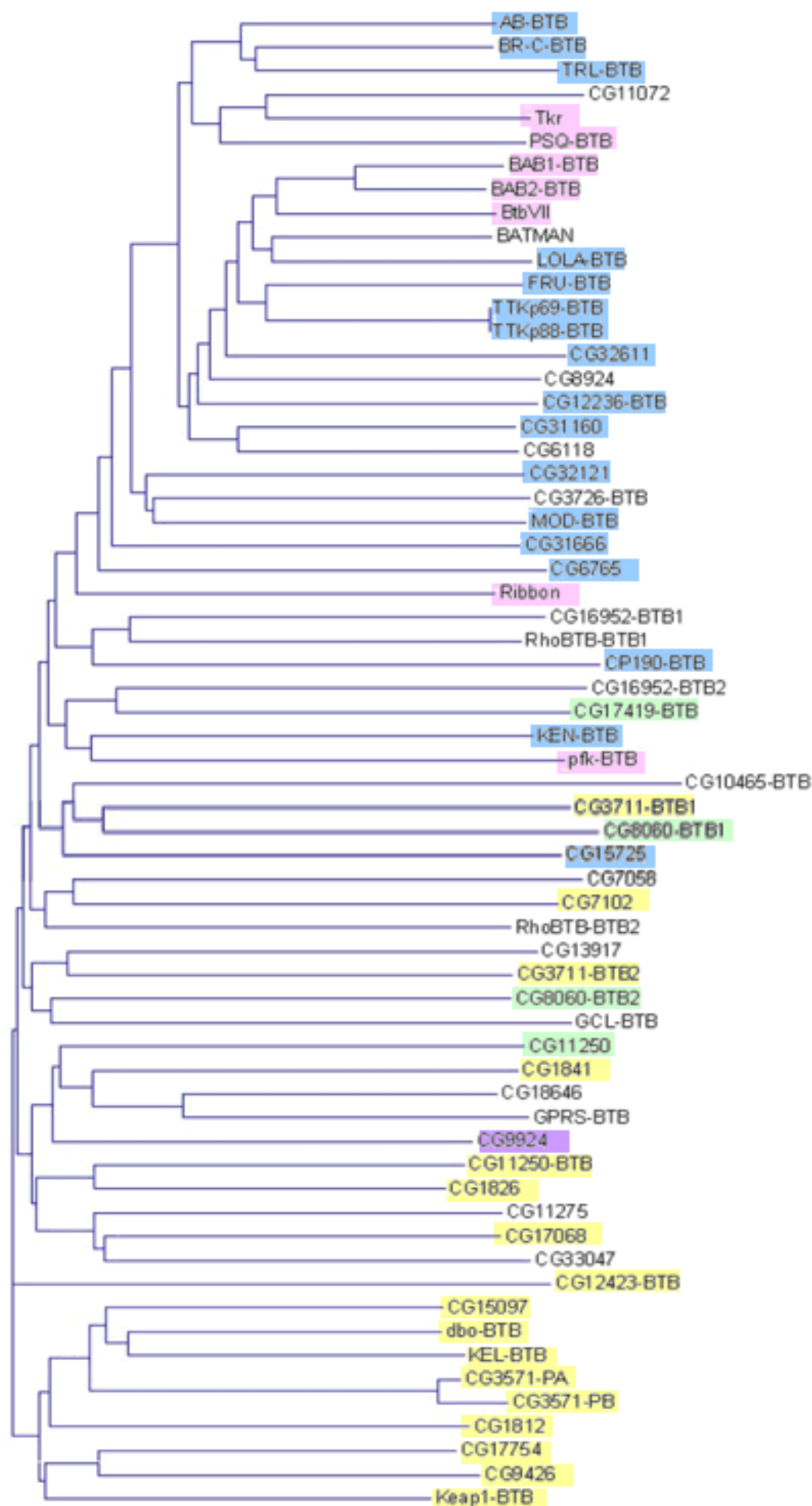

Supplement: S1 Fig — 60 BTB domain-containing proteins in the fly genome are grouped according to second conserved domains and are shaded with different colors: zinc finger in blue, Psq in pink, Ankyrin repeats in green, MATH in purple, and Kelch repeats in yellow. Those with other domains are not shaded with color. (PDF) [file pgen.1006362.s001.pdf]

# Supplementary Figure 2

A

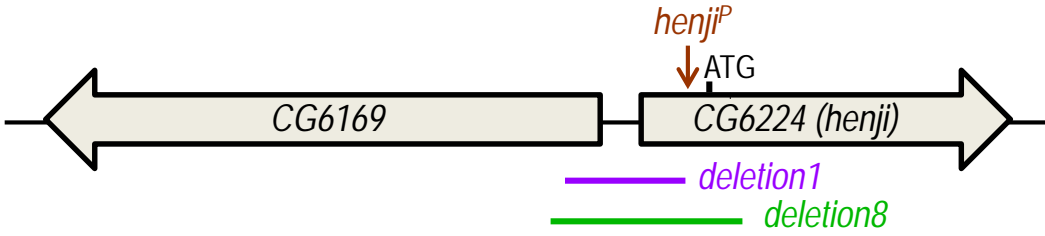

B

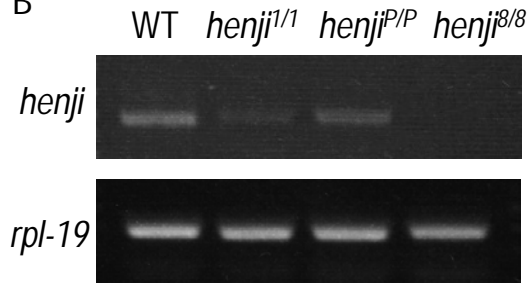

C

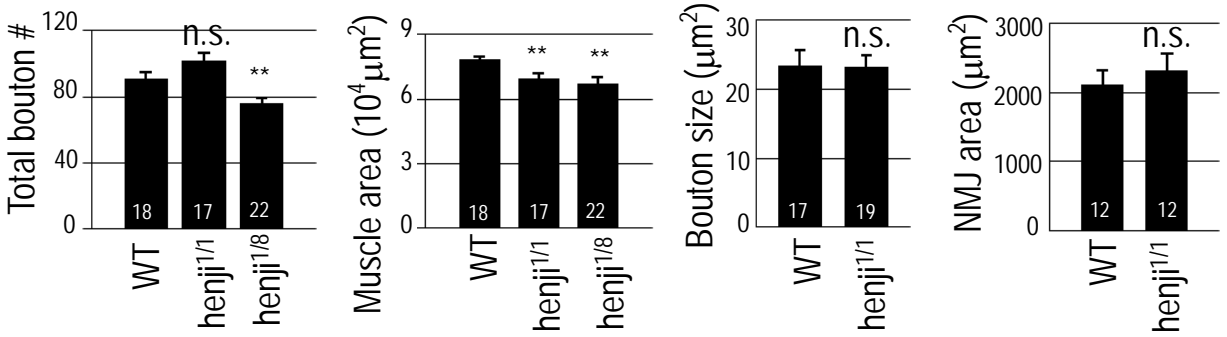

D

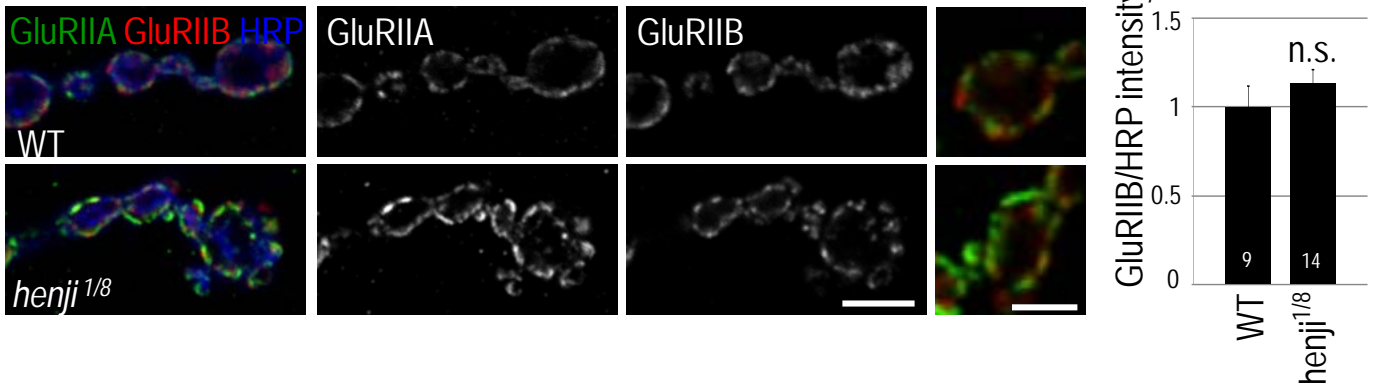

Supplement: S2 Fig — (A) Schematic diagram shows the locations of henji mutant alleles. P{GT1}Dcp2[BG01766] and P{EPg}dboHP30996 were excised to generate genomic deletions 1 and 8, respectively. deletion1 truncates part of the 5’ UTRs of the neighboring gene CG6169 and henji (CG6224). deletion8 truncates a larger part of the 5’UTR of CG6169 and the putative translation start site of henji (shown as ATG). Since these two deletion mutants also truncate parts of CG6169, they were complemented with a CG6169 genomic rescue construct in the mutant flies for phenotypic analysis. After introducing CG6169-GR, the early lethality of both deletion lines was rescued, suggesting that lethality resulted from CG6169 and that henji was a non-essential gene. The mutant carrying deletion1 or deletion8 and CG6169-GR was named as henji1 or henji8, respectively. The third mutant allele carried a PBac{PB}dboc04604 transposon insertion in the 5’UTR of henji, and this allele was named henjiP in this study. (B) RT-PCR reveals the henji mRNA levels in different mutant alleles. henji1, reducing henji gene transcription, is a strong loss-of-function allele. The hypomorphic allele henjiP shows reduced mRNA levels compared with WT. henji8, with no detectable henji mRNA, is considered a null allele. (C) Bar graph (first from left) shows total bouton numbers of both regular and satellite boutons for WT, 90.89 ± 3.69; and henji1/1, 102.00 ± 4.67; henji1/8, 75.86 ± 3.46. The second bar graph shows average muscle areas for WT, 7.84 ± 1.60; henji1/1, 6.95 ± 0.25; and henji1/8, 6.73 ± 0.28. The third bar graph shows bouton size for WT, 23.44 ± 2.34; and henji1/1, 23.56 ± 1.53. The forth bar graph shows NMJ areas for WT, 2121.69 ± 193.22; and henji1/1, 2323.85 ± 237.41. **, p < 0.01 by unpaired Student t-test. Comparisons with no significant differences (p > 0.05) are indicated by n.s. (D) Co-immunostaining of GluRIIA and GluRIIB shows the significant increase in GluRIIA intensity in henji1/8. However, the GluRIIB intensity [file pgen.1006362.s002.pdf]

Supplementary Figure 3

A

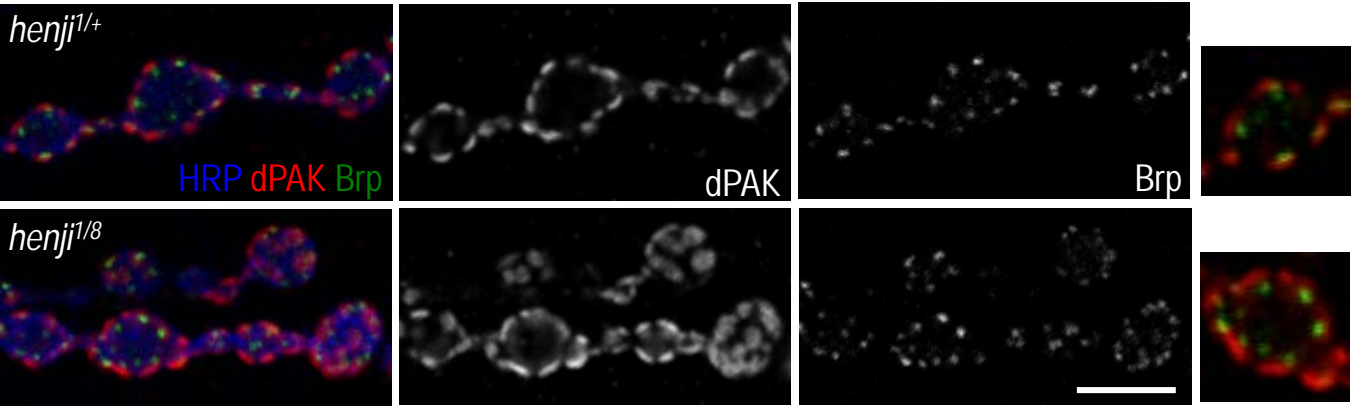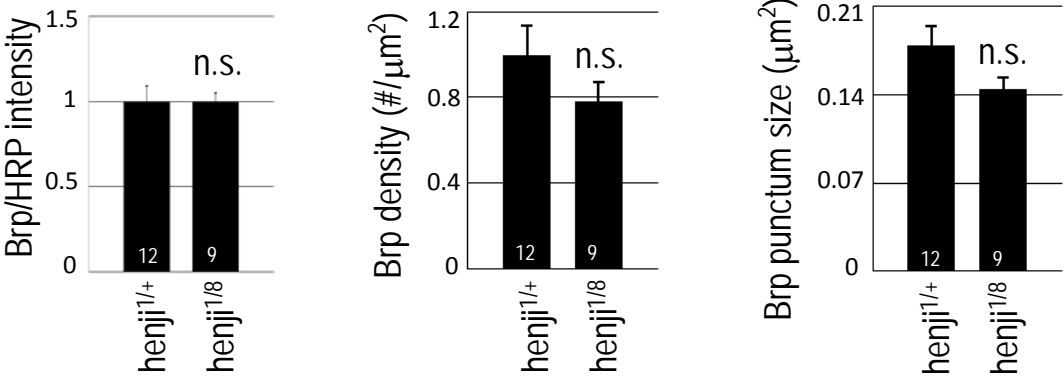

B

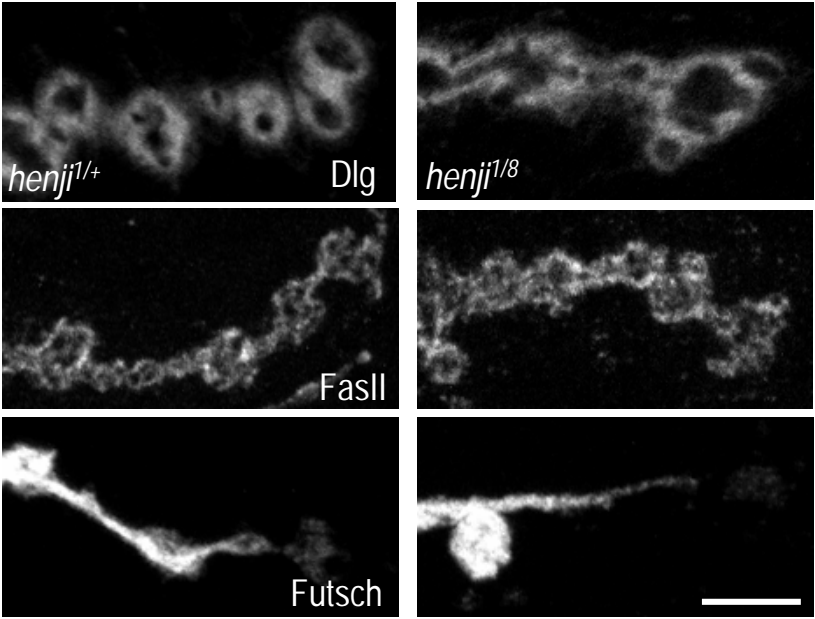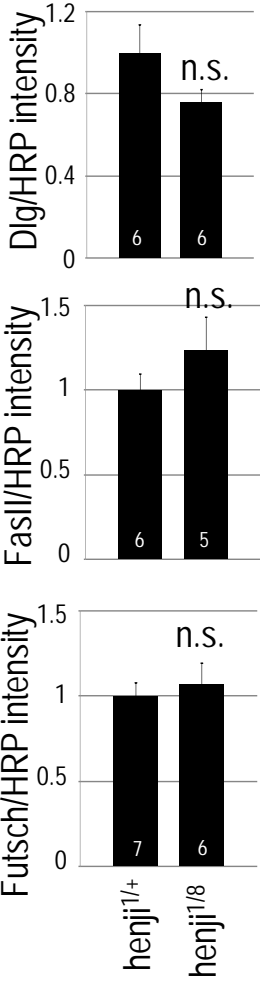

Supplement: S3 Fig — (A) Co-staining of dPAK and Brp shows dPAK accumulation in the henji1/8 mutant but Brp remains at normal levels, as compared to heterozygous henji1/+ controls. Enlarged images shows single boutons with matching Brp and dPAK puncta in both genotypes. Lower bar graphs show no significant differences (n.s.) in Brp intensity (normalized to HRP intensity), and Brp punctum density (normalized to HRP area) and size when comparing both genotypes. Brp intensity (henji1/+, 1.00 ± 0.09; henji1/8, 1.00 ± 0.05); punctum density (henji1/+, 1.00 ± 0.14; henji1/8, 0.78 ± 0.09); Brp punctum size (henji1/+, 0.18 ± 0.02; henji1/8, 0.14 ± 0.08). (B) Dlg, Futsch and FasII immunostaining show no significant differences between henji1/+ and the henji1/8. Bar graphs on the right show HRP-normalized intensities with no significant differences (n.s.) detected by unpaired Student t-test. Dlg: henji1/+, 1.00 ± 0.14; henji1/8, 0.76 ± 0.06; FasII: henji1/+, 1.00 ± 0.09; henji1/8, 1.24 ± 0.31; Futsch: henji1/+, 1.00 ± 0.08; henji1/8, 1.07 ± 0.13. Scale bar is 5 μm. (PDF) [file pgen.1006362.s003.pdf]

Supplementary Figure 4

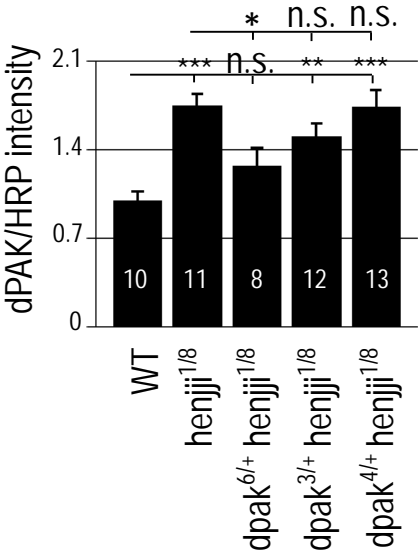

Supplement: S4 Fig — Bar graph shows the synaptic dPAK levels at NMJs that were normalized to co-stained HRP with WT set as 1. WT, 1.00 ± 0.07; henji1/8, 1.75 ± 0.09; henji1/8 dpak6/+, 1.28 ± 0.13; henji1/8 dpak3/+, 1.51 ± 0.10 and henji1/8 dpak4/+, 1.74 ± 0.13. Significance by unpaired Student t-test is shown with * for p < 0.05, ** for p < 0.01, *** for p < 0.001, and n.s. for no significance. (PDF) [file pgen.1006362.s004.pdf]

Supplementary Figure 5

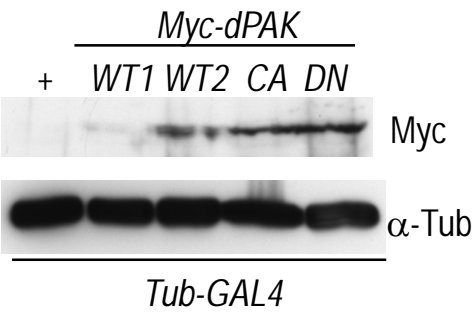

Supplement: S5 Fig — Myc-dPAK WT1, WT2, CA and DN transgenes were overexpressed ubiquitously by Tub-GAL4. Adult fly head extracts were collected for Western blot analysis by anti-Myc antibodies. Although WT1 shows a very low expression level, WT2, CA, and DN have similar expression levels. WT2 were used in this study. Immunoblotting of α-Tubulin (α-Tub) serves as a control. (PDF) [file pgen.1006362.s005.pdf]

# Supplementary Figure 6

A

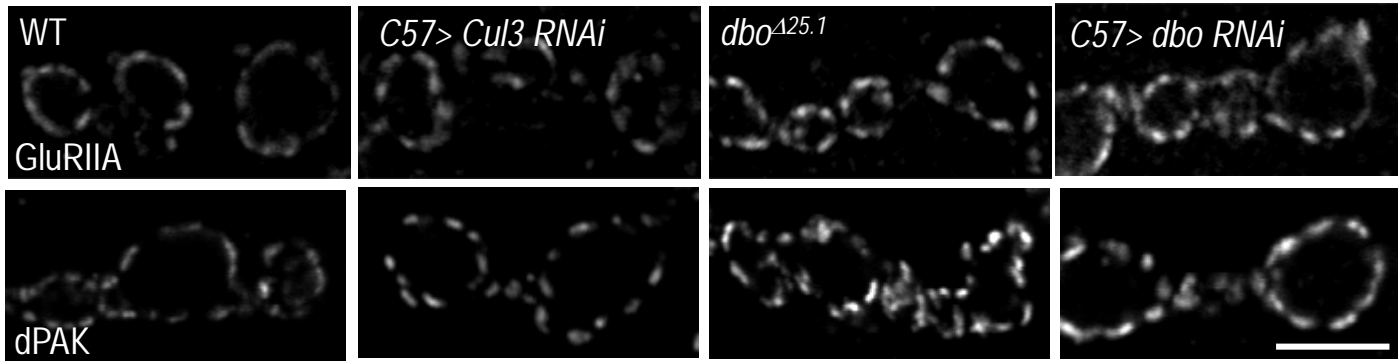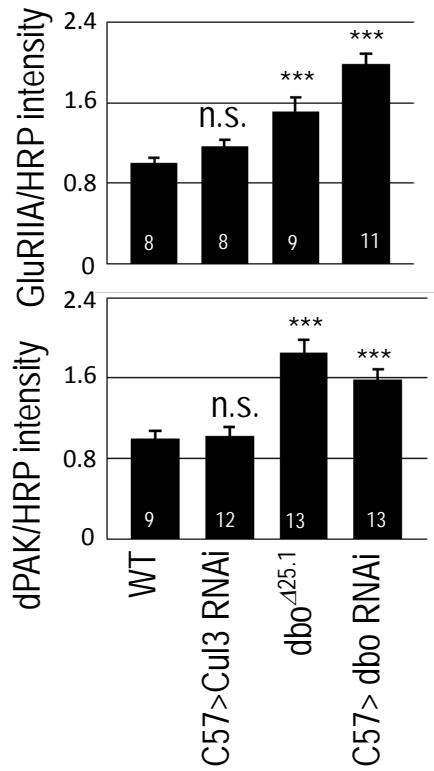

B

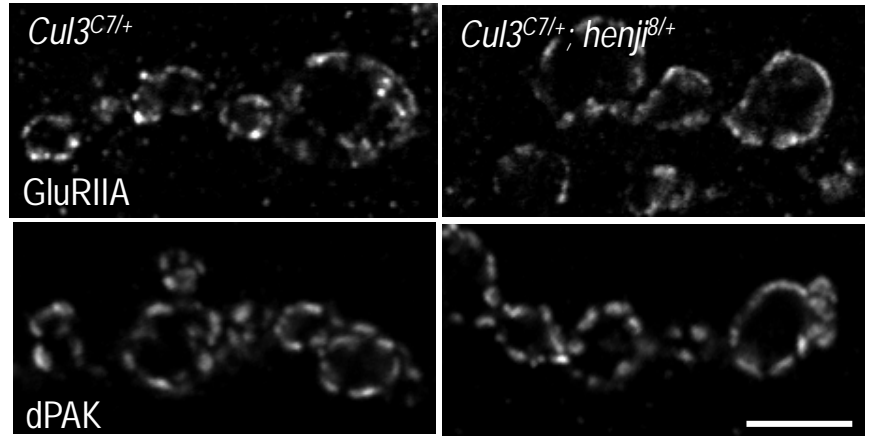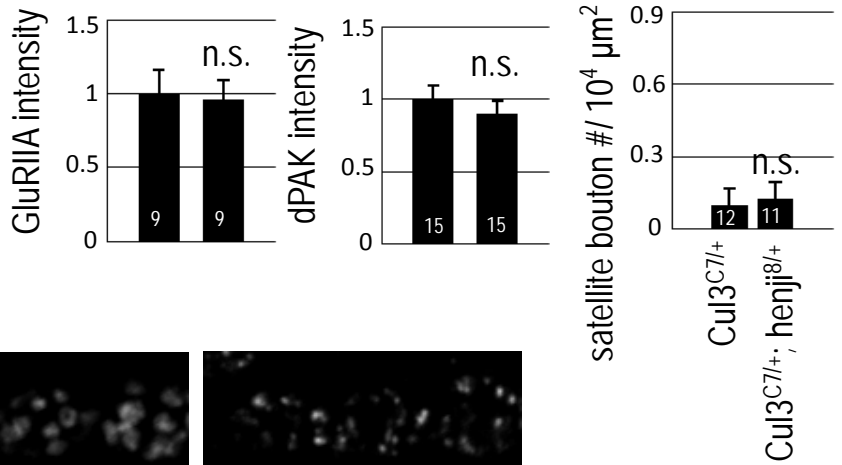

C

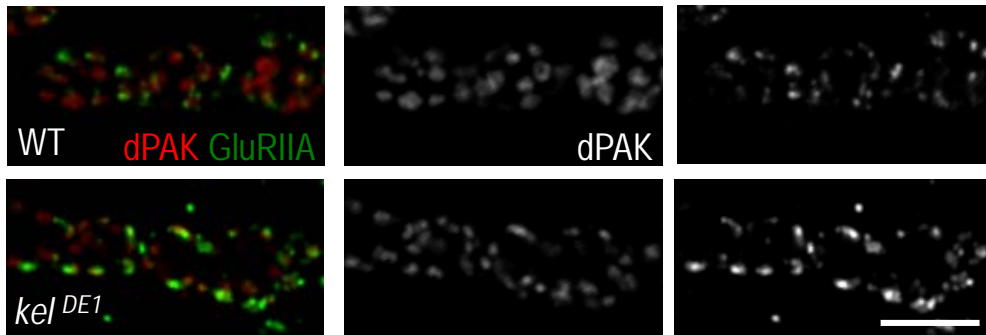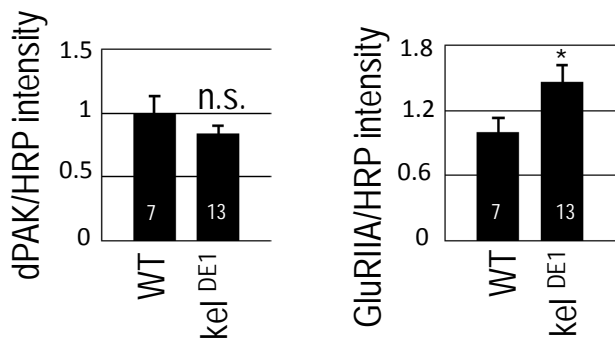

Supplement: S6 Fig — (A) Immunostaining of GluRIIA and dPAK in WT, muscle knockdown of Cul3, dbo[Δ25.1] and muscle knockdown of dbo [61]. Quantification of GluRIIA and dPAK intensities with normalization to HRP intensities and the WT value is set as 1. GluRIIA: WT, 1.00 ± 0.06; C57>Cul3 RNAi, 1.16 ± 0.07; dbo[Δ25.1], 1.98 ± 0.11, and C57>dbo RNAi, 1.51 ± 0.14. dPAK: WT, 1.00 ± 0.08; C57>Cul3 RNAi, 1.02 ± 0.09; dbo[Δ25.1], 1.85 ± 0.13 and C57>dbo RNAi, 1.59 ± 0.09. (B) Immunostaining shows no elevation of GluRIIA and dPAK levels in double heterozygous Cul3C7/+ henji8/+ mutants as compared to Cul3C7/+. Quantification of GluRIIA and dPAK intensities normalized to HRP intensities. Satellite bouton numbers are normalized to muscle areas. GluRIIA: Cul3C7/+, 1.00 ± 0.16; Cul3C7/+ henji8/+, 0.97 ± 0.12. dPAK: Cul3C7/+, 1.00 ± 0.09; Cul3C7/+ henji8/+, 0.90 ± 0.08. Satellite boutons: Cul3C7/+, 0.10 ± 0.07; Cul3C7/+, henji8/+, 0.12 ± 0.07. (C) Co-immunostaining of dPAK and GluRIIA in WT and kelDE1 homozygotes. dPAK and GluRIIA intensities were quantified and normalized to HRP intensity. dPAK: WT, 1.00 ± 0.13; kelDE1, 0.84 ± 0.06, and GluRIIA: WT, 1.00 ± 0.13; kelDE1, 1.46 ± 0.15. Significance by unpaired Student t-test is shown with * for p < 0.05, ** for p < 0.01, *** for p < 0.001, and n.s. for no significance. Scale bars are 5μm. (PDF) [file pgen.1006362.s006.pdf]

Supplementary Figure 7

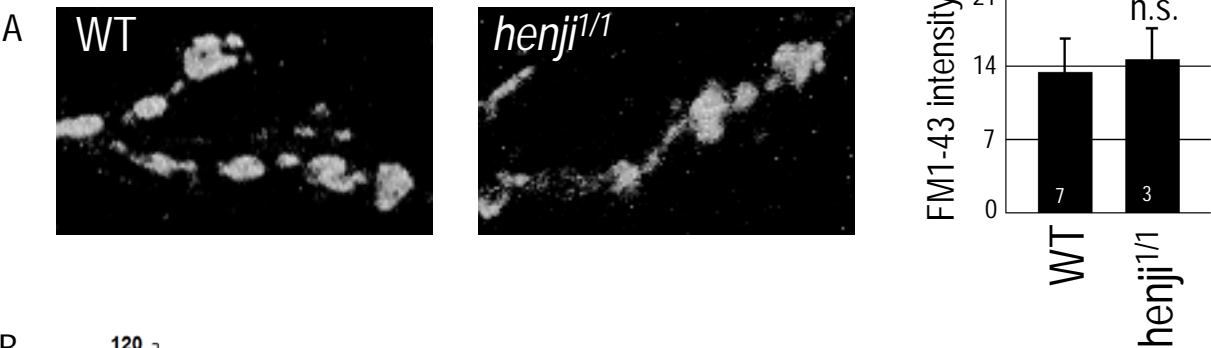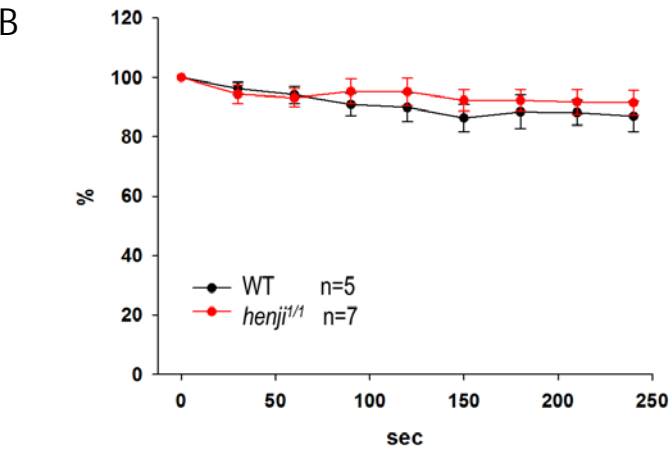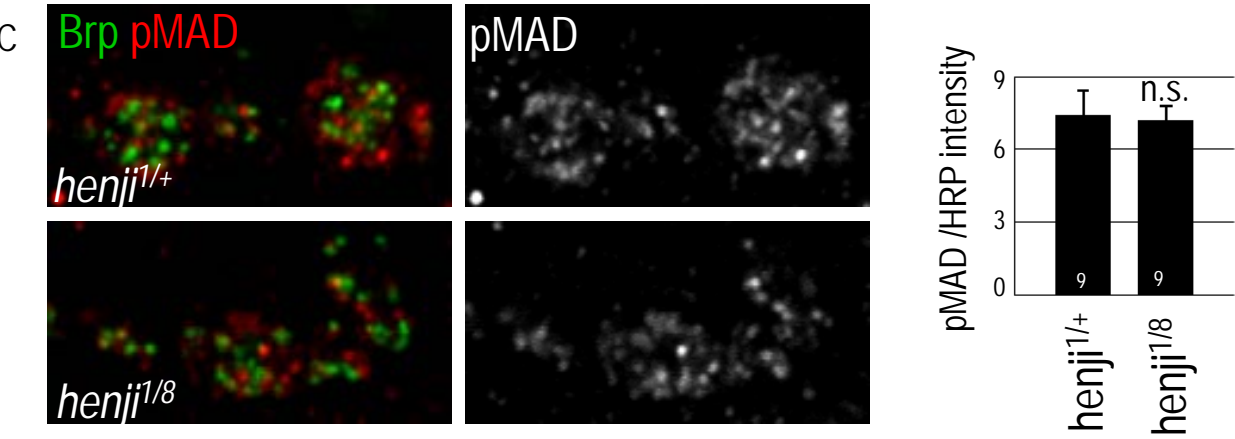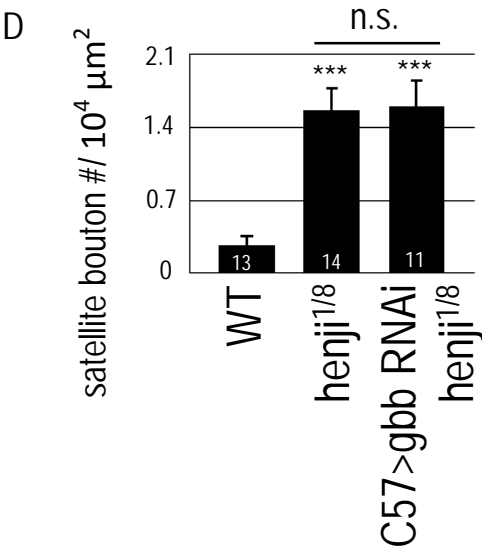

Supplement: S7 Fig — (A) FM1-43 dye uptake is used as an indicator for endocytosis in boutons of WT and henji1/1. Intensity of FM1-43 was quantified and no significant difference was detected between WT and henji1/1. FM1-43 intensity inside boutons: WT, 13.51 ± 3.23; henji1/1, 14.78 ± 2.87. Scale bar is 10 μm. (B) High-frequency stimulation in 2 mM [Ca2+]. No significant defect was found in henji mutant. (C) Immunostaining of pMAD in the henji1/+ heterozygous control and the henji1/8 mutant. The pMAD intensities were quantified and normalized to HRP intensities. henji1/+, 7.41 ± 1.01; henji1/8, 7.23 ± 0.55. No significance (n.s.) was detected between two genotypes. Scale bar is 5 μm. (D) Knockdown gbb in the postsynaptic muscle cells has no suppressing effect on satellite boutons in the henji1/8 mutant. Satellite bouton numbers were normalized to muscle areas. WT, 0.27 ± 0.09; henji1/8, 1.57 ± 0.21; C57> gbb RNAi henji1/8, 1.60 ± 0.24. *** for p < 0.001 and n.s. for no significance by unpaired Student t-test. (PDF) [file pgen.1006362.s007.pdf]
