# Supplementary material for: Dbo/Henji Modulates Synaptic dPAK to Gate Glutamate Receptor Abundance and Postsynaptic Response
Source: PLoS Genet. 2016 Oct 13;12(10):e1006362. doi: 10.1371/journal.pgen.1006362 (PMC5065118; doi:10.1371/journal.pgen.1006362)
Supplement: S1 Table — (DOCX) [file pgen.1006362.s008.docx]

**Supplementary 1 Table. Ultrastructural parameters from TEM analysis.**

|  | WT (17) | *henji^1/1^* (43) | *henji^P/P^* (17) |
| --- | --- | --- | --- |
| Bouton perimeter (μm) | 7.61±0.84 | 7.88±0.49 | 7.88±0.59 |
| Number of active zones/ bouton | 4.00±0.37 | 4.72±0.25 | 4.53±0.49 |
| Active zone length (μm) | 0.64±0.03 | 0.75±0.03** | 0.77±0.05* |
| Active zone length/bouton perimeter (%) | 34.00±2.14 | 46.15±1.91*** | 45.57±4.08* |
| Number of T-bars/ active zone | 0.35±0.06 | 0.32±0.05 | 0.33±0.05 |
| Number of vesicles bound to active zone | 4.83±1.04 | 5.07±0.77 | 3.94±0.37 |
| Number of vesicles bound to T-bar | 30.54±2.27 | 22.93±1.48** | 18.77±1.13*** |
| T-bar length (μm) | 0.18±0.02 | 0.11±0.01** | 0.17±0.02 |

Statistical significance by unpaired Student t-test is indicated with * for p < 0.05, ** for p < 0.01, and *** for p < 0.001. No significance is not indicated. Sample number for each genotype is in parenthesis.
